# Alert-QSAR. Implications for Electrophilic Theory of Chemical Carcinogenesis

**DOI:** 10.3390/ijms12085098

**Published:** 2011-08-11

**Authors:** Mihai V. Putz, Cosmin Ionaşcu, Ana-Maria Putz, Vasile Ostafe

**Affiliations:** 1 Laboratory of Computational and Structural Physical Chemistry, Chemistry Department, West University of Timişoara, Pestalozzi Street No.16, Timişoara, RO-300115, Romania; E-Mail: vostafe@cbg.uvt.ro (V.O.); 2 “Nicolas Georgescu-Roegen” Forming and Research Center of West University of Timişoara, 4th, Oituz Street, Timişoara, RO-300086, Romania; E-Mail: cosminel_ctin@yahoo.com (C.I.); 3 Institute of Chemistry Timişoara of the Romanian Academy, 24 Mihai Viteazul Bld., Timişoara, RO-300223, Romania

**Keywords:** genotoxic carcinogenesis, structural alerts, OECD principles, residual-QSAR, electronegativity and chemical hardness reactivity principles

## Abstract

Given the modeling and predictive abilities of quantitative structure activity relationships (QSARs) for genotoxic carcinogens or mutagens that directly affect DNA, the present research investigates structural alert (SA) intermediate-predicted correlations *A^SA^* of electrophilic molecular structures with observed carcinogenic potencies in rats (observed activity, A = Log[1/TD_50_], *i.e*., $A^{\mathrm{SA}}=f\left(X_{1}^{\mathrm{SA}},X_{2}^{\mathrm{SA}},\ldots \right)$). The present method includes calculation of the recently developed residual correlation of the structural alert models, *i.e.*, ${\mathrm{ARA}}^{\mathrm{SA}}=f\left(A-A^{\mathrm{SA}},X_{1}^{\mathrm{SA}},X_{2}^{\mathrm{SA}},\ldots \right)$. We propose a specific electrophilic ligand-receptor mechanism that combines electronegativity with chemical hardness-associated frontier principles, equality of ligand-reagent electronegativities and ligand maximum chemical hardness for highly diverse toxic molecules against specific receptors in rats. The observed carcinogenic activity is influenced by the induced SA-mutagenic intermediate effect, alongside Hansch indices such as hydrophobicity (LogP), polarizability (POL) and total energy (Etot), which account for molecular membrane diffusion, ionic deformation, and stericity, respectively. A possible QSAR mechanistic interpretation of mutagenicity as the first step in genotoxic carcinogenesis development is discussed using the structural alert chemoinformation and in full accordance with the Organization for Economic Co-operation and Development QSAR guidance principles.

## 1. Introduction

Chemical carcinogenesis became an experimental science in 1918, when Yamagiwa and Lchihawa reproduced coal tar carcinogenicity in rabbit skin [1]. In 1930, Yoshida reported hepato-carcinogenicity of a pure aminoazo dye in rats [2], while in 1938, Hueper *et al.* induced urinary bladder cancer in dogs using 2-naphthylamine [3]. In the following decades, the carcinogenicity of a number of polycyclic hydrocarbons, and the strong dependence of their activities on structural features, was demonstrated [4].

Thereafter, Miller and Miller suggested that chemical carcinogens are converted *in vivo* to reactive electrophilic derivates that combine with nucleophilic groups such as nucleic acids and proteins. These conclusions were based on observations that changes in genetic information were caused by reaction with alkylating electrophiles and were the initial event in chemical carcinogenesis. In this framework, then, chemical carcinogens are simply strong electrophilic reactants [5,6].

The benchmark *in vitro* model of chemical carcinogenicity is a series of genetically-engineered *Salmonella typhimurium* bacterial strains created by Bruce Ames [7]. John Ashby contributed to the identification and compilation of a list of structural alerts (SAs), which are chemically reactive functional groups that induce mutations and cancer [8]. All of the four basic sets of SAs (generically identified as Ashby SAs, Bailey SAs, Kazius SAs and the Ames test) give similar results for Salmonella mutagenicity and rodent carcinogenicity [9].

Ames (1984) showed that a high percentage of known human carcinogens can be detected as mutagens (around 83%) [7]. Oxygen radicals are the most important class of mutagens contributing to aging and cancer, but they are not detected as mutagens by the standard *Salmonella* strains used in such assays. Hydroperoxides generated by lipid peroxidation may damage DNA through the generation of hydroxyl radicals, which are also the main agents in radiation damage to DNA.

“Mutagenicity” refers to a substance’s capacity to cause genetic mutations; it is of great public concern because it is closely related to carcinogenicity and, potentially, reproductive toxicity. Mutagenicity can be assessed experimentally with the Ames *Salmonella* test, which has an estimated reproducibility of 85%. This intrinsic limitation of the *in vitro* test, along with the need for faster and less expensive predictive alternatives, has led to interest in other assessment methods, such as in silico structure-activity (toxicity) relationship [QSA(T)R] models [10].

In one example, because nitro compounds were observed to induce mutations in the *S. typhimurium* TA98 strain, QSTR techniques were employed to develop models of nitroarene mutagenicity using a set of 197 nitro-aromatic and heteroaromatic molecules. These models employed QSAR techniques with 2D and 3D descriptors such as electron distribution, spatial disposition, molecular volume, hydrophobicity, steric features, solubility and ionization constants [11].

A recent study by Perez-Garrido *et al.* (2010) investigating the relationships between various bond types and mutagenicity suggested a correlation between mutagenicity and hydrophobicity and molecular volume. This led to a series of proposed structural alerts for mutagenicity. The reactivity of carbonyl groups in electrophilic addition processes was found to be influenced by the size and electronic effects of the substituents; molecular size was found to increase with the number of hydrogens attached to sp^3^ carbons, leading to a reduction in mutagenicity; and the presence of a terminal double bond was also correlated with mutagenicity. The models created have a concordance of 86% and correctly classify 95% of mutagenic substances [12].

Investigation of the biological property of carcinogenicity using computational and network interaction studies may help to explain the increasing incidence of cancer. A successful predictive model must be generally valid for compounds with diverse molecular structures, obtain similar results for substances with similar physico-chemical properties and correctly model the relationship between sensitivity and specificity [13].

Recently, CAESAR, a project developing models of chemical properties that affect human health, included prediction of carcinogenicity in its set of goals [14,15]. Recent logical-chemoinformatics studies, using methods such as Counter Propagation Artificial Neural Networks (CP ANN), found that high levels of sensitivity (75%) and specificity (69%) were related to electrotopological states and molecular connectivity [16].

These models provide predictions of biological activity and information about the structures, toxicities and solubilities of all compounds analyzed. Since the European Parliament adopted a legislative proposal for the REACH (*registration, evaluation and authorization of chemicals*) chemical management system in 2006, *in vivo* testing has diminished in importance because the similar information can be obtained in silico through quantitative structure-activity relationships (QSARs) [17]. Carcinogens can be separated into two classes based on their mechanisms of action: genotoxic carcinogens directly damage DNA (thus producing mutagenesis as the first step of chemical carcinogenesis) [9,13], while epigenetic carcinogens do not bind covalently to DNA.

A (Q)SAR model, according to Organization of Economic Cooperation and Development (OECD) guidelines, has the following characteristics: a defined endpoint, an unambiguous algorithm, a defined domain of applicability, statistical performance and predictive power, and a mechanistic interpretation [18,19]. In this sense, a valid model will be the simplest and most mechanistically transparent. Good statistical performance is not sufficient for selection of a model; it should be compatible with existing knowledge in the fields of QSAR, chemistry and biology [9]. In construction of the model, if the compounds considered are not sufficiently similar in chemical-biological space or do not have a predefined activity ordering (e.g., a Gaussian distribution), the model will have poor predictive value for smaller datasets. Construction of a QSAR requires experimental data, molecular representation and fitting algorithms. Different QSAR implementations have varying requirements due to resource constraints and legal considerations; any model must therefore be evaluated in the context of its intended use [9].

The present work makes two contributions for advanced residual-QSAR modeling of genotoxic carcinogenesis [20]. First, to gain mechanistic insight into mutagenesis, the influence of explicit reactivity and electrophilic parameters on the derived QSAR models of carcinogenesis was investigated. Second, the residual QSAR method is extended by considering molecular fragment information in structural alerts, which aids in elucidating the electrophilic theory of chemical carcinogenesis.

## 2. Alert-QSAR Method

It was recently shown [20] that direct and residual correlations should be combined; that is, for a given parameter set and an observed endpoint set, $\left({\left\{X_{i}\right\}}_{i=\bar{1,M}},A\right)$, the direct QSAR can be written as

(1) $$A^{M}=a_{0}+\sum_{i=1}^{M}b_{0i}X_{i}$$

and residual analysis gives

(2) $${\mathrm{ARA}}^{M}=a_{1}+b_{1}\left(A-A^{M}\right)$$

Here the superscript “*M*” refers to either the full molecule or the structural parameters for each molecule under study. Equations (1) and (2), with the assumption that the obtained residual-QSAR matches the observed activity,

(3) $$A={\mathrm{ARA}}^{M}$$

provide the “*asymptotic residual QSAR*”,

(4) $${\mathrm{ARA}}^{M}=\frac{1}{1-b_{1}}\left[a_{1}-b_{1}a_{0}-b_{1}\sum_{i=1}^{M}b_{0i}X_{i}\right]$$

Because Equation (4) is modulated by the parameters *b*_1_ → 1, it is invariantly obtained throughout this procedure regardless of the structural parameters or form of the direct QSAR. While such behavior is common in cancer modeling, it presents conceptual limitations in assessing the model of interest.

The present approach avoids such drawbacks by making use of the properties of the structural alerts. It specifically employs the physico-chemical properties of these alerts to build associated QSARs and residual QSAR counterparts to build a multi-regression model of the molecular mechanism.

**Figure 1.**
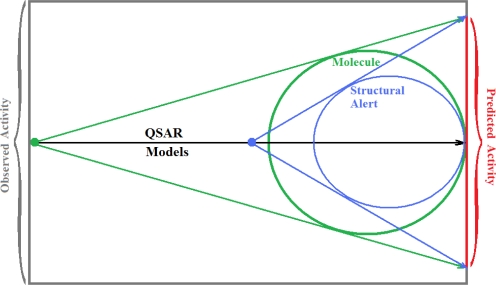
The *alert-QSAR method* uses structural alerts to assemble a molecular fragment QSAR model that has predictive power similar to that of full molecular modeling.

The present alert-QSAR algorithm is shown qualitatively in Figure 1. This algorithm provides activity predictions either by considering the full molecular structures or the substructures of the structural alerts.

The algorithm assumes a structure–activity multi-linear correlation problem using the structural alert (SA) parameters and observed endpoint set: $\left({\left\{X_{i}^{\mathrm{SA}}\right\}}_{i=\bar{1,M}},A\right)$. The associated *alert-QSARs* corresponding to specific regressions over subsets of the structural parameters may be computed, for instance, for (1 = *m*_1_ < … < *m_i_* < *m_i_* _+ 1_ < … < *m_j_* _– 1_ < *m_j_* < … < *m_M_* = *M*):

(5) $$A_{\left(m_{i},m_{j}\right)}^{\mathrm{SA}}=a_{0\left(m_{i},m_{j}\right)}^{\mathrm{SA}}+\sum_{k=m_{i}}^{m_{j}}b_{0k}^{\mathrm{SA}}X_{k}^{\mathrm{SA}}$$

The residuals associated with Equation (5) are

(6) $${\mathrm{RA}}_{\left(m_{i},m_{j}\right)}^{\mathrm{SA}}=A-A_{\left(m_{i},m_{j}\right)}^{\mathrm{SA}}$$

Equations (5) and (6) give the formed residual-alert-QSAR equation

(7) $${\mathrm{ARA}}_{\left\{\left(m_{i},m_{j}\right)\right\}}^{\mathrm{SA}}=a_{1\left\{\left(m_{i},m_{j}\right)\right\}}^{\mathrm{SA}}+\sum_{m_{i}=1m_{j}}^{m_{j-1}}\sum_{=m_{i+1}}^{m_{k}\leq M}b_{1\left(m_{i},m_{j}\right)}^{\mathrm{SA}}{\mathrm{RA}}_{\left(m_{i},m_{j}\right)}^{\mathrm{SA}}$$

Combining this expression with the activity matching condition of Equation (3), here rewritten in actual terms,

(8) $$A={\mathrm{ARA}}_{\left\{\left(m_{i},m_{j}\right)\right\}}^{\mathrm{SA}}$$

yields the structural alert residual correlation.

(9) $${\mathrm{ARA}}_{\left\{\left(m_{i},m_{j}\right)\right\}}^{\mathrm{SA}}=\frac{1}{1-\sum_{m_{i}=1m_{j}}^{m_{j-1}}\sum_{=m_{i+1}}^{m_{k}\leq M}b_{1\left(m_{i},m_{j}\right)}^{\mathrm{SA}}}\left[a_{1\left\{\left(m_{i},m_{j}\right)\right\}}^{\mathrm{SA}}-\sum_{m_{i}=1m_{j}}^{m_{j-1}}\sum_{=m_{i+1}}^{m_{k}\leq M}b_{1\left(m_{i},m_{j}\right)}^{\mathrm{SA}}\left(a_{0\left(m_{i},m_{j}\right)}^{\mathrm{SA}}+\sum_{k=m_{i}}^{m_{j}}b_{0k}^{\mathrm{SA}}X_{k}^{\mathrm{SA}}\right)\right]$$

Equation (9) improves upon Equation (4) by using structural alert information instead of molecular information, avoiding singularities in the denominator, *i.e.*,

(10) $$1\neq \sum_{m_{i}=1m_{j}}^{m_{j-1}}\sum_{=m_{i+1}}^{m_{k}\leq M}b_{1\left(m_{i},m_{j}\right)}^{\mathrm{SA}}$$

This gives a residual-alert QSAR, a self-consistent correlation equation for the observed activity based on structural alert-predicted activities and residuals thereof. The method is next illustrated by reanalyzing previous toxicological carcinogenic series and studies [20].

## 3. Results on Genotoxic Carcinogenesis

**Figure 2.**
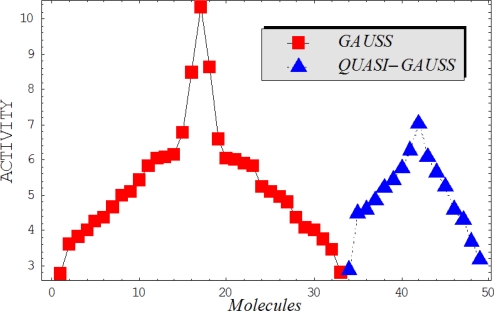
Graphical representation of the working activities for molecules in Tables 1 and 2, classified under the “Gaussian” and “quasi-Gaussian” series for the training and testing QSARs, respectively.

This study targeted carcinogenic activity in rats (*Rattus norvegicus*), as measured by TD_50_ values (in *mg/kg body wt/day*) derived from the Carcinogenic Potency Database (CPD) [16]. Activity is expressed here as a function of the TD_50_ values, A = Log(1/TD_50_). The working series of molecules, were chosen to have a high diversity molecular structure and fulfilling the Topliss-Costello rule [21] according to which their cardinal should be at least 5-times larger the number of structural descriptors used. They are separately shown in Tables 1 and 2, as calibration/trial/trainin sets using Gaussian screening and as test set using quasi-Gaussian distribution screening (Figure 2), respectively. The parameters recommended by Hansch [22] (hydrophobicity, polarizability and total energy) and special reactivity indices (electronegativity and chemical hardness), all computed using the semiempirical PM3 method, were used for both full molecules and structural alerts for the molecules found in Tables 1 and 2.

However, as noted by Hansch, “there is no substitute for extensive experience…in physical organic chemistry and QSAR” [22]. Highly diverse molecular groups were employed in assessing the observed genotoxic carcinogenesis/mutagenicity. Several particular choices or “degrees of freedom” can be considered in to bring the analysis in line with the traditional QSAR dogma of “congeneric molecules”.

- *Physicochemical parameters*: meaningful physicochemical parameters as hydrophobicity, polarizability, total energy, electronegativity, and chemical hardness should be considered in order to better interpret the derived models in terms of molecular mechanisms.
- *Universal hydrophobicity*: when a full molecule is identical to its structural alert, the sign of the structural alert may be flipped relative to that of the molecule for the action-reaction solubility characteristics. For instance, this can be applied to the LogP of the reagent because it is the logarithm (base 10) of the partition coefficient (P), the ratio of the compound’s organic (oil)-to-aqueous phase concentrations. Therefore, the opposite and equal values of the molecule and its identical structural alert induce a kind of “universality” in the solvation ability of the concerned toxicant. This approach may be applied to molecules with a recognized high toxicological or carcinogenic potential, and should not be overestimated in the molecular series employed. For the present trial series (Table 1), this approach was employed for molecule no. **3**, acetaldehyde (ethanal, C_2_H_4_O). Such an approach is justified, because this compound’s average global production is about 10^6^ tons/year [23]; it is a common electrophile in organic synthesis [24] (in agreement with Miller’s electrophilic theory [5,6] of genotoxic carcinogenesis: “there is *sufficient* evidence for the carcinogenicity of acetaldehyde (the major metabolite of ethanol) in experimental animals”) [25]; it is a probable carcinogen in humans [26], but occurs naturally in coffee, bread and ripe fruit, and is produced by plants as part of their normal metabolism; and it can be spread through air, water, land or groundwater pathways and can be absorbed through inhalation, smoking or consumption [27].
- *Equal steric properties*: molecules with similar carcinogenic properties may be considered to have equal optimized stericities, *i.e.*, total energies, when their true values are in the same domains. Thus, non-carcinogenic molecules may be considered to be similar in some of their physicochemical properties, including stericity (in this case, associated with total energy). For instance, in the trial series of compounds (Table 1) molecules **8** and **10**, have energies of −94064.03906 [eV] and −108827.09 [eV], respectively (as calculated with PM3 and geometry optimization). These can be considered to have the same stericities in intra-cellular binding, due to their similar energies, similar activities in rats (as given by the CPD) [28], close positions in the Gaussian graph (Figure 2) and their identical carcinogenic characteristics [29] such as damage factors, disease-specific factors, and the same uncertainty factor for the combined damage and effect factors. Consequently, the common value was set from the more carcinogenic molecule (**10**). However, as is the case with the above “universal hydrophobicity” adjustment, the equal stericity principle should be applied with caution (as a rule, it should be applied to less than 10% of the molecules in a series) and only to mark non-congeneric series of molecules with similarity physicochemical properties.

The direct and residual-QSARs as applied to the full molecules and structural alerts shown in Tables 1 and 2 were applied as follows:

***Step I***: *Structural alert QSARs* for the trial compounds of Table 1 gave the structural alert activities *A^SA^* shown in Table 3. Full molecular QSARs for the trial molecules of Table 1 are reported in Table 4 as “M” computed/predicted models. Combined QSAR predictions based on the molecular descriptors from Table 1 and the structural alert activities of Table 3 are reported in Table 4 as “*M∧SA*”; these results showcase how consideration of the structural alerts allows systematic improvement of the predictions over the molecular indicators. Note that the structural alert parameters may be combined with the molecular ones only at the level of full molecules; in this way, full molecular parameters are combined with predicted activity at the molecular level as provided by structural alerts modeling.

***Step II***: *Residual QSARs* for the structural alert models derived in *Step I* are computed, considering the predicted activities in Table 3; the results are presented in Table 5. Considerable correlation was found, indicating the indirect influence of structural alerts on mutagenicity and carcinogenesis.

***Step III***: *Structural residual alert QSARs* were obtained by selecting models from *Step II* that reproduce the structural alerts’ parameter correlations (10) as given in Equation (9). The residual-alert methodology may lead to new equations besides those presented in Table 3. The results are displayed in Table 6, with correlation performances reported for the trial molecules of Table 1 and the test compounds of Table 2.

***Step IV***: *Euclidean paths for residual-alert QSARs* for the trial molecules of Table 1 and the test compounds of Table 2 were constructed from the models of *Step III.* The models were arranged so that each model emerges from the previous one on the basis of their common descriptors; the results are reported in Table 7 by employing the Euclidean path between two successive QSAR models (computed endpoints),

(11) $$\left[{\mathrm{QSAR}}^{\left[a\right]},{\mathrm{QSAR}}^{\left[b\right]}\right]=\sqrt{{\left(R_{\left[a\right]}-R_{{\left[b\right]}^{\prime }}\right)}^{2}}$$

***Step V***: *Optimum paths for residual-alert QSARs* were derived from the results of *Step IV* by searching the minimum paths and the associated hierarchy according to the formal constraint [31–35]

(12) $$\delta \left[{\mathrm{QSAR}}^{\left[1\right]},\ldots {\mathrm{QSAR}}^{\left[k\right]}\ldots ,{\mathrm{QSAR}}^{\left[m\right]},\right]=0$$

where *QSAR*^[1]^,...*QSAR*^[^*^k^*^]^,...,*QSAR*^[^*^m^*^]^ represent the endpoint residual-QSAR regression models computed with 1, …, *k*, …, *m*≤*M* structural parameters, respectively. These paths were computed for both trial and test compounds, and their average values (Table 7) for the residual-alert and direct-alert models (Table 6) are reported. The average column of Table 7 shows two sets of first (alpha), second (beta) and third (gamma) pathways in the ergodic pathways [36], *i.e*., those uniquely contained QSAR models across all possible combinations, namely:

- those based on *residual-alert QSARs*:
  (13a) $$\begin{array}{c} \begin{array}{c} \alpha :I_{a}\to {\mathrm{II}}_{a}^{2}\to III_{2}\\ \begin{array}{c} \chi^{\mathrm{SA}},E_{\mathrm{tot}}^{\mathrm{SA}} \end{array}\ldots \begin{array}{c} \chi^{\mathrm{SA}},{\mathrm{LogP}}^{\mathrm{SA}},E_{\mathrm{tot}}^{\mathrm{SA}} \end{array}\ldots \begin{array}{c} \chi^{\mathrm{SA}},{\mathrm{POL}}^{\mathrm{SA}},{\mathrm{LogP}}^{\mathrm{SA}},E_{\mathrm{tot}}^{\mathrm{SA}} \end{array} \end{array} \end{array}$$
  (13b) $$\begin{array}{c} \begin{array}{c} \beta :I_{d}\to {\mathrm{II}}_{b}^{3}={\mathrm{II}}_{d}^{1}\to {\mathrm{III}}_{5}\\ \begin{array}{c} \eta^{\mathrm{SA}},E_{\mathrm{tot}}^{\mathrm{SA}} \end{array}\ldots \begin{array}{c} \eta^{\mathrm{SA}},{\mathrm{POL}}^{\mathrm{SA}},E_{\mathrm{tot}}^{\mathrm{SA}} \end{array}\ldots \begin{array}{c} \chi^{\mathrm{SA}},\eta^{\mathrm{SA}},{\mathrm{POL}}^{\mathrm{SA}},E_{\mathrm{tot}}^{\mathrm{SA}} \end{array} \end{array} \end{array}$$
  (13c) $$\begin{array}{c} \gamma :I_{e}\to {\mathrm{II}}_{b}^{2}={\mathrm{II}}_{e}^{2}\to {\mathrm{III}}_{3}\\ \begin{array}{c} {\mathrm{POL}}^{\mathrm{SA}},{\mathrm{LogP}}^{\mathrm{SA}} \end{array}\ldots \begin{array}{c} \eta^{\mathrm{SA}},{\mathrm{POL}}^{\mathrm{SA}},{\mathrm{LogP}}^{\mathrm{SA}} \end{array}\ldots \begin{array}{c} \eta^{\mathrm{SA}},{\mathrm{POL}}^{\mathrm{SA}},{\mathrm{LogP}}^{\mathrm{SA}},E_{\mathrm{tot}}^{\mathrm{SA}} \end{array} \end{array}$$
- and those based on *direct-alert QSARs:*
  (14a) $$\begin{array}{c} \alpha :I_{g}\to {\mathrm{II}}_{c}={\mathrm{II}}_{d}^{2}\to {\mathrm{III}}_{3}\\ \begin{array}{c} {\mathrm{LogP}}^{\mathrm{SA}},E_{\mathrm{tot}}^{\mathrm{SA}} \end{array}\ldots \begin{array}{c} \eta^{\mathrm{SA}},{\mathrm{LogP}}^{\mathrm{SA}},E_{\mathrm{tot}}^{\mathrm{SA}} \end{array}\ldots \begin{array}{c} \eta^{\mathrm{SA}},{\mathrm{POL}}^{\mathrm{SA}},{\mathrm{LogP}}^{\mathrm{SA}},E_{\mathrm{tot}}^{\mathrm{SA}} \end{array} \end{array}$$
  (14b) $$\begin{array}{c} \beta :I_{a}\to {\mathrm{II}}_{a}^{2}\to {\mathrm{III}}_{2}\\ \begin{array}{c} \chi^{\mathrm{SA}},E_{\mathrm{tot}}^{\mathrm{SA}} \end{array}\ldots \begin{array}{c} \chi^{\mathrm{SA}},{\mathrm{LogP}}^{\mathrm{SA}},E_{\mathrm{tot}}^{\mathrm{SA}} \end{array}\ldots \begin{array}{c} \chi^{\mathrm{SA}},{\mathrm{POL}}^{\mathrm{SA}},{\mathrm{LogP}}^{\mathrm{SA}},E_{\mathrm{tot}}^{\mathrm{SA}} \end{array} \end{array}$$
  (14c) $$\begin{array}{c} \gamma :I_{d}\to {\mathrm{II}}_{b}^{3}={\mathrm{II}}_{d}^{1}\to {\mathrm{III}}_{5}\\ \begin{array}{c} \eta^{\mathrm{SA}},E_{\mathrm{tot}}^{\mathrm{SA}} \end{array}\ldots \begin{array}{c} \eta^{\mathrm{SA}},{\mathrm{POL}}^{\mathrm{SA}},E_{\mathrm{tot}}^{\mathrm{SA}} \end{array}\ldots \begin{array}{c} \chi^{\mathrm{SA}},\eta^{\mathrm{SA}},{\mathrm{POL}}^{\mathrm{SA}},E_{\mathrm{tot}}^{\mathrm{SA}} \end{array} \end{array}$$

The remaining issue is to decide among these two pathways, while noting, for instance, the α- and v-residual-alert-QSARs are reproduced as β- and γ-direct-alert-QSARs, respectively. These and the resulting molecular mechanisms for the actual genotoxic effects on rats are clarified within the OECD-QSAR principles, as discussed below.

## 4. OECD-QSAR Principles Discussion

Generally, risk assessment comprises hazard identification (a qualitative risk assessment dealing with the inherent toxicity of a chemical substance), qualitative mutagenicity assessment (how likely an agent is to be a human mutagen), quantitative mutagenicity risk assessment (how much mutational damage is likely under particular exposure scenarios), dose-response assessment (relationship between the dose of a chemical and adverse effects) and exposure assessment (populations exposed to toxic chemicals).

Chemicals that have exhibited mutagenic activities in various test systems have been found in foods, tobacco, drugs, food additives, cosmetics, industrial compounds, pesticides and consumer products [37]. In this context, the OECD-QSAR principles can be used to guide any quantitative risk assessment of the carcinogenic potential under study through the provided QSAR models. They will be reviewed and illustrated using the present case study while emphasizing the specific advancements elucidated in this work.

### 4.1. Principle 1: A Defined Endpoint

According to OECD guidance, “the intent of QSAR Principle 1 (*defined endpoint*) is to ensure clarity in the endpoint being predicted by a given model, since a given endpoint could be determined by different experimental protocols and under different experimental conditions. It is therefore important to identify the experimental system that is being modeled by the (Q)SAR”. Note that the actual endpoint, the genotoxic carcinogenesis with mutagenesis as the first step of organism cells’ apoptosis [9,13], arises in principle with the same binding mechanism as binding/breaking DNA, through a group with high diverse structures, giving rise to the following updating QSAR end-point approaches:

- *(Eco-) toxicological studies*, having various end-points (such as inhibition, activation, death, sterility, irritations, *etc.*) yet produced by a group of similar molecules, *i.e.*, the case of *congeneric studies*;
- and *carcinogenic studies*, having essentially the same end-point as the exacerbated apoptosis that in principle diffuses in the organism no matter what the initial point of triggering is, may be initiated by highly structurally diverse molecule, being therefore classified as *non-congeneric studies*.

While the first case above is usually treated by ordinary (or direct) QSAR approaches, the second category is less frequently treated with the central QSAR dogma of congenericity. It therefore requires special approaches, such as the recent study [20] and actual residual-QSAR modeling. This relies on the fact that if no direct high correlation can be found, then there is a high probability that the action is residual, complementary or indirect.

### 4.2. Principle 2: An Unambiguous Algorithm

According to the OECD guidance, the intent of QSAR-Principle 2 (unambiguous algorithm) is to ensure transparency in the predictive algorithm. The actual alert-QSAR method is outlined in Section 2, and Hansch physico-chemical parameters were used in the implementation:

- *hydrophobicity* (LogP), corresponding to trans-cellular membrane diffusion and with *translation motion* of the molecules;
- *polarizability* (POL), accounts for the dipole perturbation and ionic interaction, and is associated with the *vibrational motion* of the molecules in organism; it further accounts for potentially electrophilic effects that triggers cancer, according with the Millers’ theory [5,6], and sustained by the recent research [20]; and
- *optimal total energy* (Etot), which contains steric information about the molecule’s 3D structure since it is given by the equilibrium conformation [30]; it may serve therefore as a potential for the *rotational motion* of the molecules when triggered by interaction with organism’s receptor.

These three structural parameters that encode information about the basic classical molecular are based on the quantum structural computation. However, for the present QSAR for chemical carcinogenicity, additional reactivity indices such as electronegativity (**χ**) and chemical hardness (*η*) were considered. These indices, by definition, relate to the first and second derivatives (or changes/variations) of the total energy respecting the total number of electrons, and describe the effects of donating (through ionization potential IP and HOMO levels) and accepting electrons (through electronic affinity EA and LUMO levels), within the frozen core approximation of Koopmans’ theorem [38].

(15) $$\chi ={\left(\frac{\partial E_{N}}{\partial N}\right)}_{V(r)}≅\frac{\left(E_{N_{0}-1}-E_{N_{0}})+(E_{N_{0}}-E_{N_{0}+1}\right)}{2}\equiv \frac{\mathrm{IP}+\mathrm{EA}}{2}≅-\frac{{ɛ}_{\mathrm{LUMO}}+{ɛ}_{\mathrm{HOMO}}}{2}$$

and

(16) $$\eta =\frac{1}{2}{\left(\frac{\partial^{2}E_{N}}{\partial N^{2}}\right)}_{V(r)}≅\frac{E_{N_{0}+1}-2E_{N_{0}}+E_{N_{0}-1}}{2}=\frac{\mathrm{IP}-\mathrm{EA}}{2}≅\frac{{ɛ}_{\mathrm{LUMO}}-{ɛ}_{\mathrm{HOMO}}}{2}$$

This enables a qualitative understanding of the basic phenomena within the so-called *chemical orthogonal space*–COS (χ ⊥ *η*); the first quantity Equation (15) is associated with the mid-level between the HOMO and LUMO energies, while the second quantity in Equation (16) gives the HOMO-LUMO interval/gap [39]. This idea combines the orthogonality of *χ* and *η* (necessary for QSAR analysis) with the associated reactivity principles described below [40].

**Figure 3.**
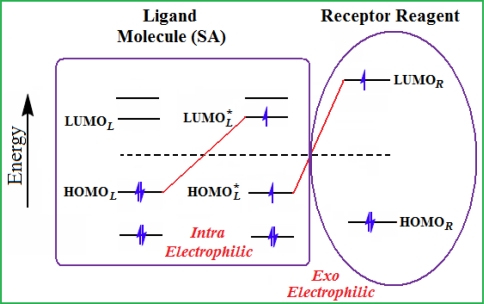
The electrophilic docking structure-reactivity algorithm correlating electronegativity and chemical hardness with chemical carcinogenesis. The algorithm starts with electronegativity docking (equalization) between the ligand and the receptor (the middle dashed line). Next, intra-molecular (in connection with specific structural alerts) maximization of the HOMO-LUMO gap (*i.e*., of chemical hardness) is accomplished by exo-electrophilic transfer of an electron from ligand to receptor.

- *The electronegativity equalization (EE) principle* relies on the equivalence of the negative electronegativity from Equation (15) with a system’s chemical potential [41], fulfilling the Gibbs rule of phases between two molecular states. The EE principle was originally stated by Sanderson as “the molecules in their fundamental state, the electronegativities of different electronic regions in molecule–are equal” [42]. This principle was further generalized and applied to many-electron systems [43]. In this work, the principle is applied at the level of ligand-receptor binding (Figure 3). The molecular electronegativity is first equalized with that of the receptor, leading to the selection of the molecular fragment (structural alert) with electronegativity complementary to that of the receptor or adjustment of the receptor’s pocket that to fit with the ligand electronegativity. This stage corresponds to a sort of *electronegativity based docking* based on the fundamental quantum EE principle. The induced interaction is then stabilized through chemical hardness.
- *The maximum hardness principle* derives from Pearson’s observation that “there seems to be a rule of nature that molecules (or the many-electronic systems in general, n.a.) arrange themselves (in their ground or valence states, n.a.) to be as hard as possible” [44]. This principle, which has been quantitatively justified [45–48], stipulates that a maximum HOMO-LUMO gap is associated with a stabilized interaction for a molecular sample.

In this study, these two fundamental reactivity principles involve *intra-electrophilic* (*intramolecular*) electron transfer from the HOMO and LUMO of the ligand molecule (or SA), such that, after donating one HOMO electron to the molecular or SA-LUMO, *exo-electrophilic* (*intermolecular*) electron transfer between the new molecule or SA-HOMO* and receptor LUMO occurs. This leaves a larger SA-HOMO–LUMO gap through ligand HOMO* relaxation (which is formally removed, so the gap between LUMO* and the second order ligand’s HOMO is increased). This produces an overall *electrophilic docking* effect (Figure 3). Note that other electrophilic mechanisms involving molecular or SA HOMO and LUMO frontier transformations and relaxations may be possible, but the two stages of electronegativity equalization and chemical hardness maximization should be equally satisfied.

Together, the electronegativity and chemical hardness indices unambiguously describe a ligand-receptor docking mechanism via intra- and exo-electrophilic stages, generalizing the Millers’ theory of direct electronic transfer between the molecular or SA HOMO and receptor LUMO [6].

### 4.3. Principle 3: A Defined Domain of Applicability

OECD guidance justifies the need to define an applicability domain (Principle 3) by the fact that (Q)SARs are reductionist models with inevitable limitations. These include limitations in terms of the types of chemical structures, physicochemical properties and mechanisms of action for which the models can generate reliable predictions. This principle is inherently linked with the first OECD-QSAR endpoint criterion. However, in the present carcinogenicity study, it acquires a further degree of freedom because no particular molecular structure is required, though the same mechanism and endpoint should be eventually provided.

As such, the molecules in Tables 1 and 2 span many organic classes and derivatives, including amides, amines, aromatic systems, lactones, nitrites, quinines, cyanides, urethanes, ketones and cycloalkanes. From a given pool of molecules, the trial and testing series may be selected through Gaussian or quasi-Gaussian screening (Figure 2). Such a procedure may eventually generalize the previous central dogma of QSAR [9] because it may be applied either to congeneric and non-similar structural molecules while focusing on ordering their observed activities in a naturally or normal statistical series, while they are associated with essentially the same interaction mechanism towards the receptor [20].

About 10% of the trial pool of molecules may be transformed in their hydrophobicities and total optimized energies to acquire universal or equal properties depending on their uses or evident similarly recorded activity effects, respectively (see Section 3).

### 4.4. Principle 4: Appropriate Measures of Goodness-of-Fit, Robustness and Predictivity

OECD QSAR principle 4 (appropriate measures of goodness-of–fit, robustness and predictivity) makes a distinction between the *internal performance of a model* (as represented by goodness-of-fit and robustness or the correlation within the trial set of molecules) and *the predictivity of a model* (as determined by external validation on a test set of molecules).

The external test set of molecules is the preferred option in QSAR validation because it assures the reliability of a given derived model. This type of validation also includes situations in which the test sets provide better statistics than the calibration series, generalizing the analytical course [49]. In Step V of the alert-QSARs of Table 7, both the trial and test Euclidean paths were considered; they were thereafter averaged in order to employ information from the statistical performances and predictions of both the trial and test sets.

Note that the Euclidean distance may be further enriched with other statistical outputs and factors, although all directly or indirectly depend on the correlation factor; we are considering such enrichment [50].

### 4.5. Principle 5: A Mechanistic Interpretation

The intent of OECD QSAR Principle 5 is not to reject models that have no apparent mechanistic basis but to ensure that some consideration is given to the possibility of a mechanistic association between the descriptors used in a model and the endpoint being predicted and to ensure that this association is documented. Since the physico-chemical QSAR parameters were chosen in this study, a mechanistic interpretation of the models is possible. This nevertheless follows specific steps, some of them integrated in the previously discussed OECD-QSAR principles.

**Figure 4.**
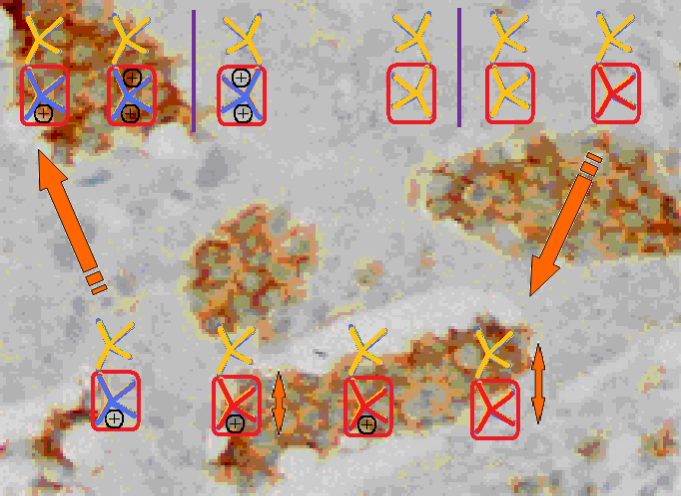
Illustration on a ligand-receptor cyclic interaction coordinate of the molecular mechanism of genotoxic carcinogenesis as given by the residual-alert-QSAR correlation-path hierarchy of Equations (13a)–(13c) then summarized in Equation (17). The mechanism is superimposed over an immunohistochemical analysis of paraffin-embedded sections of rat intestinal cancer using the Caspase-2 antibody [51]. In these evolving molecular graphs (the SA region is circumvented), steric movement is represented by mirroring, electronegativity docking by changing SA colors, diffusion by translation arrows; polarizability by vibration arrows, and electrophilic docking (the final stage including the maximum hardness principle) by positive charging.

- With the alert-QSAR models, in either residual or direct forms, (Table 6), Euclidean measures between all computed models that successively that fall along the pathways of Table 7 are constructed (see also the Step IV of Section 3).
- The first optimum paths are selected on the ergodic basis, as described in Step V of Section 3 above, by applying Equations (11) and (12) for the residual and direct alert-QSARs, respectively.
- The two classes of paths (Equations (13) and (14)) are compared on the basis of their electrophilic-docking (sub)-mechanisms identified within the unambiguous algorithm stage of the second OECD-QSAR principle. Comparison of the alpha-paths of the two alert-QSAR routes reveals that only residual-alert-QSAR correctly displays the involvement of the electronegativity in docking. As a consequence, the electrophilic-docking mechanistic interpretation of genotoxic carcinogenesis will be based only on the residual-alert-QSARs; this confirms the recent assessment of residual-QSAR as the in silico modeling technique best suited for treating chemical carcinogenesis [20]. The present approach generalizes this in two ways: by detailing the mechanistic scenario with the electronegativity-to-chemical hardness reactivity-stability influence, and by considering the structural alert information in QSAR modeling rather than working with the entire molecular structural information.
- The explicit mechanistic scenario is based on the information contained within Equations (13a)–(13c), which gives rise to a natural sequence that makes a *closed loop* over all three main interactions paths, given by
  (17) $$\begin{array}{c} \alpha -\mathrm{Steric}\;\mathrm{movement}\;\begin{array}{c} E_{\mathrm{tot}}^{\mathrm{SA}} \end{array}\to \alpha -\mathrm{Electronegativity}\;\mathrm{Docking}\;\begin{array}{c} \chi^{\mathrm{SA}} \end{array}\to \alpha -\mathrm{Cellular}\;\mathrm{diffusion}\\ \begin{array}{c} {\mathrm{LogP}}^{\mathrm{SA}} \end{array}\to \alpha -\mathrm{Polarizability}\;\mathrm{movement}\;\begin{array}{c} {\mathrm{POL}}^{\mathrm{SA}} \end{array}\to \beta -\mathrm{Electrophilic}\;\mathrm{docking}\;\begin{array}{c} \eta^{\mathrm{SA}} \end{array}\to \\ \beta -\mathrm{Polarizability}\;\mathrm{movement}\;\begin{array}{c} {\mathrm{POL}}^{\mathrm{SA}} \end{array}\to \beta -\mathrm{Electronegativity}\;\mathrm{Docking}\;\begin{array}{c} \chi^{\mathrm{SA}} \end{array}\to \gamma -\mathrm{cellular}\\ \mathrm{diffusion}\;\begin{array}{c} {\mathrm{LogP}}^{\mathrm{SA}} \end{array}\to \gamma -\mathrm{Electrophilic}\;\mathrm{docking}\;\begin{array}{c} \eta^{\mathrm{SA}} \end{array}\to \gamma -\mathrm{Steric}\;\mathrm{movement}\;\begin{array}{c} E_{\mathrm{tot}}^{\mathrm{SA}} \end{array} \end{array}$$
  which is formally represented in Figure 4.

The cycle of Equation (17) provides insight into the residual looping mechanism of the molecule or structural alert; receptor interaction, especially for electrophilic docking, here was related to electronegativity and chemical action, as compared with the previous global molecular studies which were limited to Hansch parameters only [20]. During one such interaction loop, the SA-molecule acquires a charge of +2, thus entering the next electrophile-nucleophile interaction loop with even more reactivity; this eventually leads to amplified biological activity manifested by exacerbated apoptosis due to breaking newly formed bonds in DNA. This is in close agreement with Millers’ observation (see Introduction) [6].

One can go further by choosing the first five instead of the first three interaction paths from the data of Table 7, because this number is the cardinal of the employed correlation parameters in actual residual-alert-QSARs. However, though electronegativity and chemical hardness are closely related to the total energy (Equations (15) and (16)), using only the first three interacting residual-alert pathways seems appropriate for the present purpose. For future studies, the extra index of electrophilicity [52] can be also explicitly incorporated to test its conceptual value in the electrophilic theory of chemical carcinogenesis [53].

## 5. Conclusions

The carcinogenesis process is divided into three operational stages: initiation, promotion and progression, with own unique mechanisms and QSAR characteristics that lead to an integrated approach with all the important elements to be considered. Consequently, the key to successful and meaningful QSAR formulation is the selection of appropriate molecular descriptors. It is critical to minimize the appearance of false negative results to increase regulatory acceptance of the developed models [9].

For carcinogenicity, it is important to address the mechanism of action and a negative (Q)SAR prediction for genotoxicity as well as negative results of an *in vitro* test with the conditions that mechanisms are sufficiently defined. QSAR models focus on the relationship between molecular structure and biological activity based on a comparison between the activities and structures of a series of chemicals. Nevertheless, until recently, the central dogma of QSAR asserted that the more local (specific, or a con-generic series) a model is in chemical-biological space, the higher the accuracy of its predictions [9]. As such, alongside the logical computational methods used for the QSAR modeling, *i.e*., genetic algorithms (optimization and search problems), artificial neural networks (non-parametric adaptive models), self organizing maps, support vector machines (classification and regression), partial least squares, decision forests, multiple linear regressions, k-mean clustering and TSAR [54], *conceptual-QSAR* has been developed to produce the meaningful physico-chemical models and paths to interpret ecotoxicological effects [31–36]. This approach ultimately ends in modeling of the carcinogenesis of highly diverse chemical structures [20], while considering the outliers’a analysis will lead with essential non-linear correlations [55].

The present work continues these efforts with the aim of clarifying the electrophilic mechanism of genotoxic carcinogenesis by modeling intermediate steps using structural alerts of the involved toxicants. We draw several main conclusions, as follows:

- mutagenicity may be regarded as an electrophilic ligand-receptor interaction mechanism of covalent binding between the ligand molecule or SA and receptor;
- electronegativity and chemical hardness are crucial parameters in modeling the ligand-receptor interaction due to the electronegativity equalization and maximum chemical hardness principles, respectively;
- residual-QSAR is again shown to be reliable [20] in its treatment of genotoxic carcinogenesis, as it better incorporates electronegativity and chemical hardness principles across the optimally selected pathways of organism cells’ apoptosis;
- structural alert or molecular fragment analysis improves the residual-QSAR analysis with an enriched class of QSAR models that may be associated with molecular mechanisms of interaction in complex media;
- in general, the test performances are lower than the calibration models, but also models with considerably better behavior for test molecules compared with trials are found, especially when hydrophobicity and sometimes polarizability and/or reactivity parameters of chemical hardness and electronegativity are involved; this is not surprising since they fully support the cellular transduction process (LogP) jointly with electrophilic effects (stated by reactivity principles and polarizability).
- the mechanism of carcinogenesis, being activated by non-congeneric compounds, allows consideration of similar parameters at molecular level, by advancing the universal hydrophobicity and equal stericity transformations of about 10% of the trial compounds, but only in the situations in which the molecules display identical observed carcinogenic activities.

The present study may be extended to assess the best molecular/structural alerts for residual-QSARs from a given pool of compounds. As well, it can be extended to include more statistical factors, such as standard error of estimation, Fisher and Student tests, to generalize the actual Euclidean measure to more elaborated statistical paths [56]. It may be also combined with 3D docking protocols to further validate the present residual-alert quantum-mechanical structural-reactivity analysis.

## Figures and Tables

**Table 1.** Molecules from the Gaussian training set (Figure 2) and corresponding rat TD_50_ toxicities (in mg/kg body wt/day) [16] and activities A = Log(1/TD_50_) using semi-empirical PM3-computed (Hyperchem [30]) structural parameters: hydrophobicity (LogP), polarizability (POL) [Ǻ^3^], total optimized energy (Etot) [kcal/mol], electronegativity (χ = −0.5(ɛ_LUMO_ + ɛ_HOMO_)) [eV], and chemical hardness (η = 0.5(ɛ_LUMO_ – ɛ_HOMO_)) [eV].

**No.**	**Full Molecule:** **Chemical Structure**	**Full Molecule:** **Name** **Formula** **(CASRN)**	**Toxicity:** **TD_50_** **Activity:** ***A = Log[1/TD****_50_****]***	**Structural Alert (SA)**	**LogP:** **Full Molecules** ***Structural Alert***	**POL [Ǻ^3^]:** **Full Molecules** ***Structural Alert***	**Etot [kcal/mol]:** **Full Molecules** ***Structural Alert***	**χ[eV]:** **Full Molecules** ***Structural Alert***	**η[eV]:** **Full Molecules** ***Structural Alert***
1.	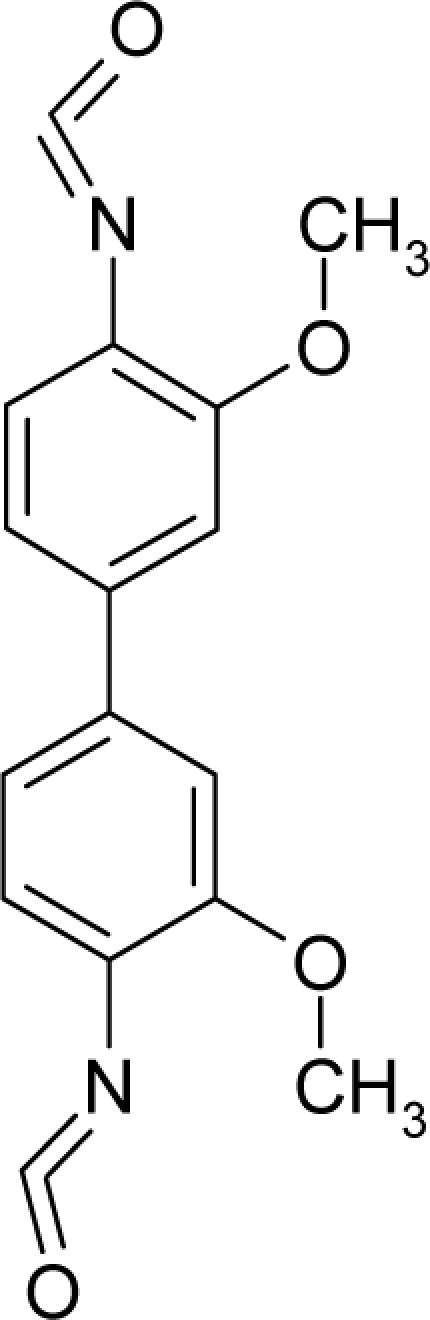	3,3′-Dimethoxy-4,4′-biphenylene diisocyanate (3,3′-Dimethoxybenzidine-4,4′-diisocyanate) C_16_H_12_N_2_O_4_ (91-93-0)	1630 *2.79*	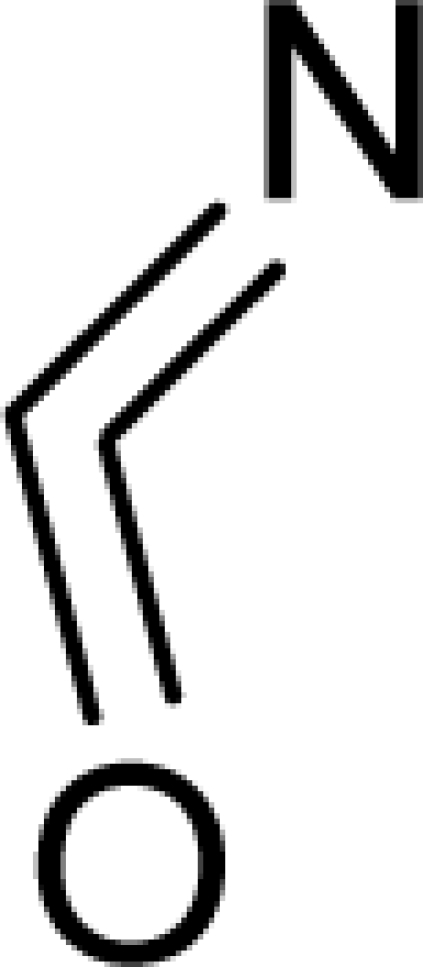	2.07 *−0.46*	30.03 *3.27*	−82478.58594 *−13584.68848*	4.74077805 *5.0497393*	3.85074395 *5.54064075*
2.	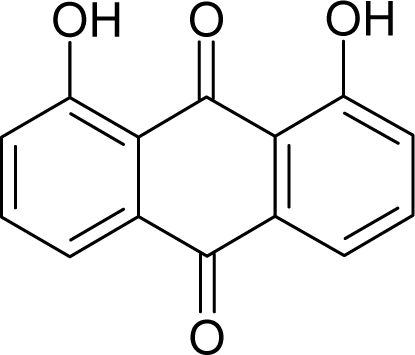	Chrysazin (Danthron) C_14_H_8_O_4_ (117-10-2)	245 *3.61*	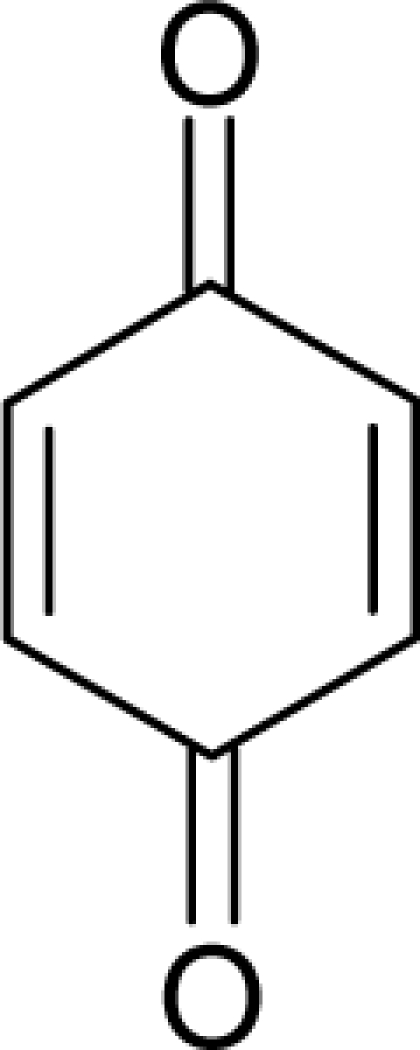	1.87 *1.52*	24.44 *10.8*	−68162.28125 *−31325.97266*	5.4079765 *6.3137325*	3.9369375 *4.6074975*
3.	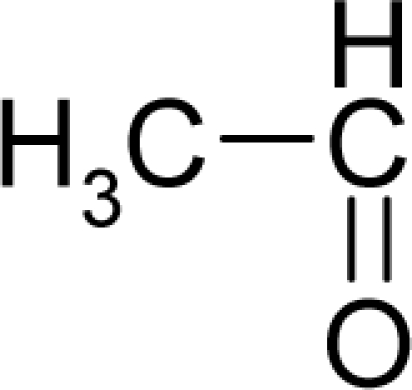	Acetaldehyde C_2_H_4_O (75-07-0)	153 *3.82*	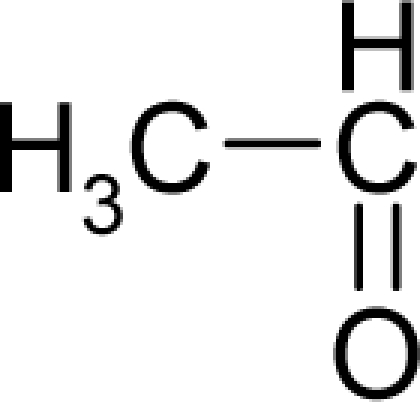	−0.58 *+0.58*	4.53 *4.53*	−13662.00781 *−13662.00781*	4.94880425 *4.94880425*	5.75505575 *5.75505575*
4.	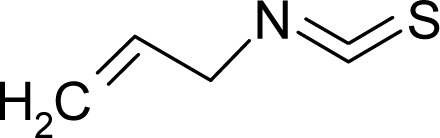	Allyl isothiocyanate C_4_H_5_NS (57-06-7)	96 *4.02*	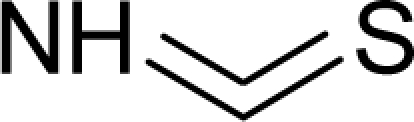	1.17 *0.19*	11.74 *6.43*	−20700.27344 *−11094.80273*	4.9388987 *5.032356*	4.2117593 *4.346452*
5.	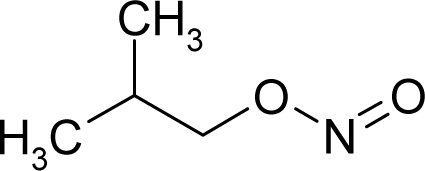	Isobutyl nitrite C_4_H_9_NO_2_ (542-56-3)	54.1 *4.27*	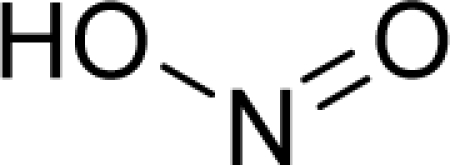	1.63 *0.38*	9.96 *2.62*	−31363 *−17580.39258*	5.294418075 *5.4457523*	5.263031925 *5.3349777*
6.	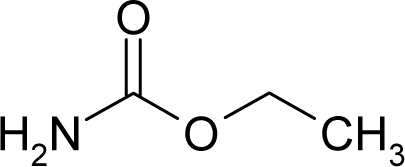	Urethane C_3_H_7_NO_2_ (51-79-6)	41.3 *4.38*	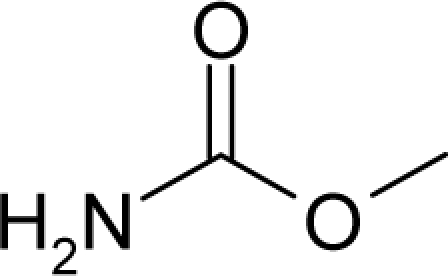	−0.06 *−0.44*	8.35 *4.68*	−27989.58203 *−21103.80273*	4.573154 *4.474373*	5.741656 *5.576267*
7.	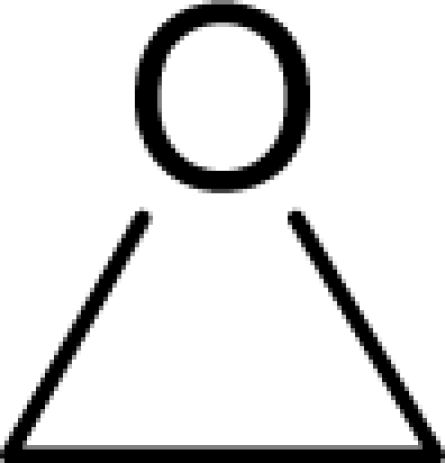	Ethylene oxide C_2_H_4_O (75-21-8)	21.3 *4.67*	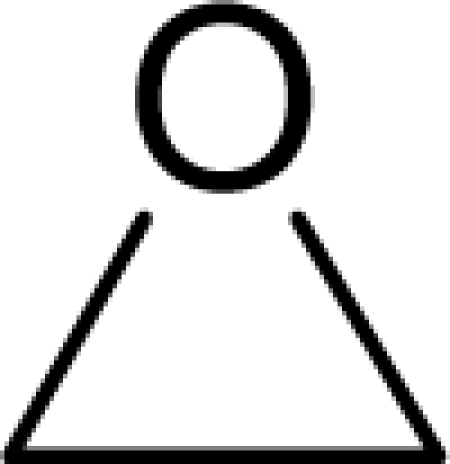	−0.16 *−0.16*	4.31 *4.31*	−13626.54297 *−13626.54297*	4.4747555 *4.4747555*	6.8617045 *6.8617045*
8.	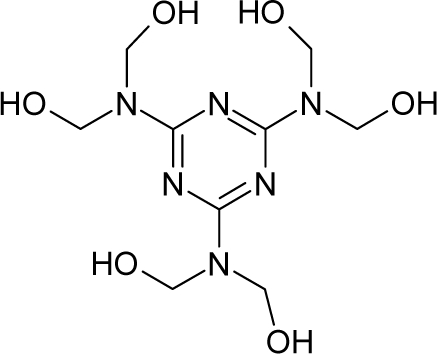	Hexa(hydroxymethyl)mela mine C_9_H_18_N_6_O_6_ (531-18-0)	10.2 *4.99*	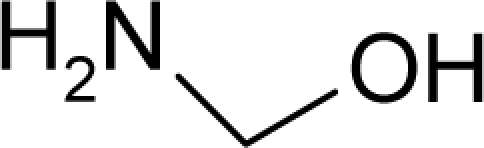	1.96 *0.08*	27.19 *5.08*	−108827.09 *−14382.44336*	4.05956015 *3.782247*	4.50969485 *7.116263*
9.	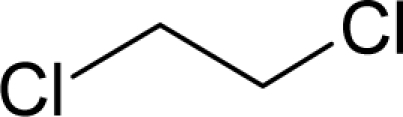	1,2-Dichloroethane C_2_H_4_Cl_2_ (107-06-2)	8.04 *5.09*	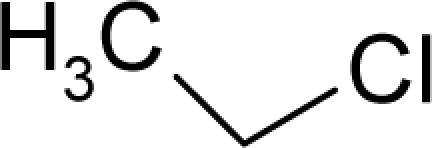	1.59 *1.22*	8.3 *6.37*	−21506.41406 *−14559.67578*	5.0714835 *4.586889*	5.6050665 *5.823301*
10.	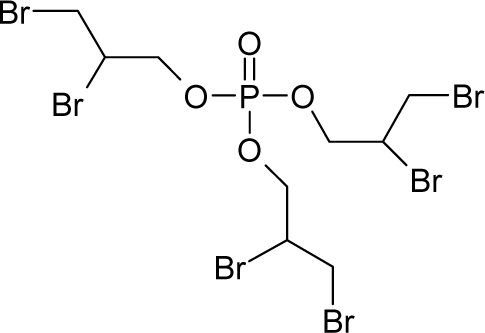	Tris(2,3-dibromopropyl) phosphate C_9_H_15_Br_6_O_4_P (126-72-7)	3.83 *5.42*	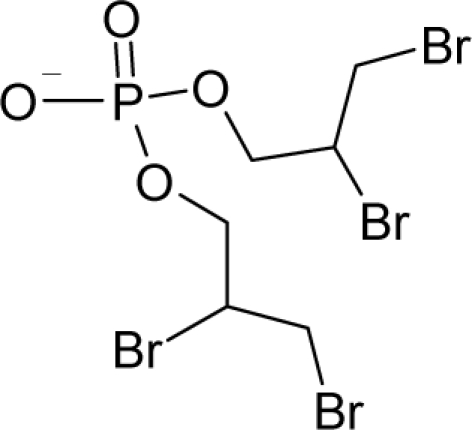	5.37 *3.73*	35.91 *25.15*	−108827.09 *−8*2903*.73*	5.6512295 *5.3925243*	4.5231705 *4.63098575*
11.	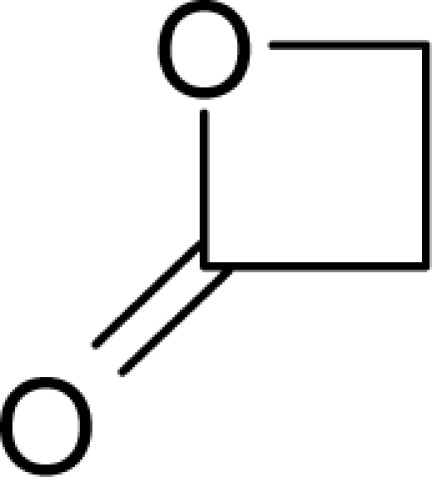	Beta-Propiolactone C_3_H_4_O_2_ (57-57-8)	1.46 *5.84*	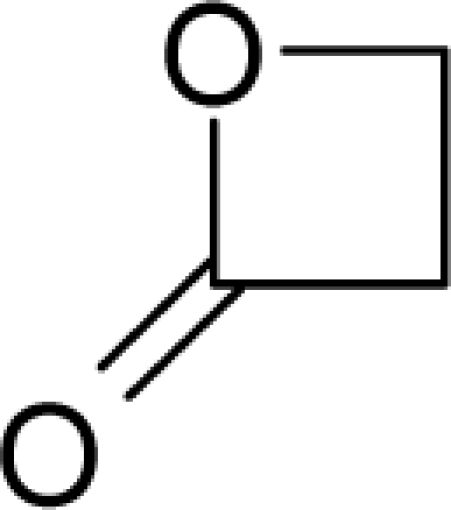	−0.25 *−0.25*	6.23 *6.23*	−23148.73047 *−23148.73047*	5.2018966 *5.2018966*	6.0842834 *6.0842834*
12.	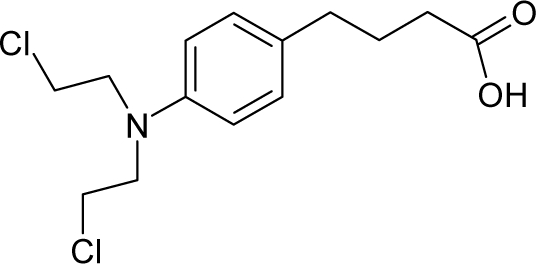	Chlorambucil C_14_H_19_Cl_2_NO_2_ (305-03-3)	0.896 *6.048*	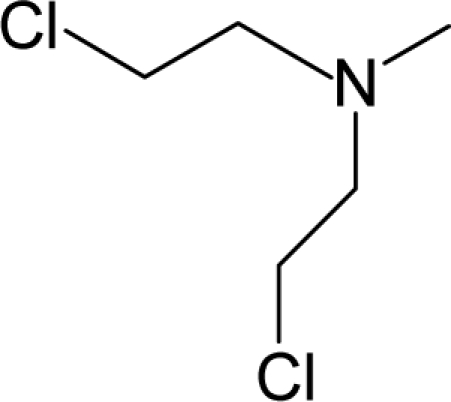	4.14 *1.2*	31.04 *13.32*	−76933.42969 *−32495.47656*	4.350258535 *4.4258064*	4.405313465 *5.31470165*
13.	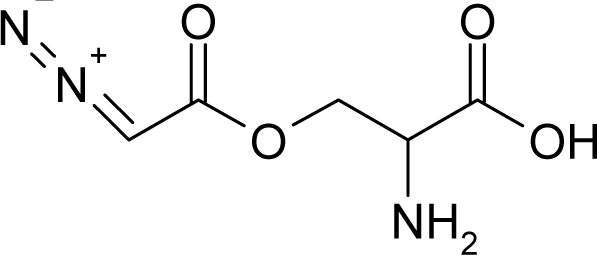	Azaserine C_5_H_7_N_3_O_4_ (115-02-6)	0.793 *6.10*	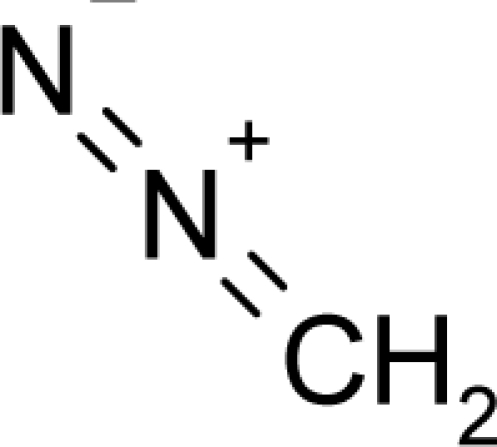	−1.03 *−0.04*	14.25 *4.11*	−54439.625 *−10877.61426*	5.2656847 *4.431137*	4.7215543 *4.794258*
14.	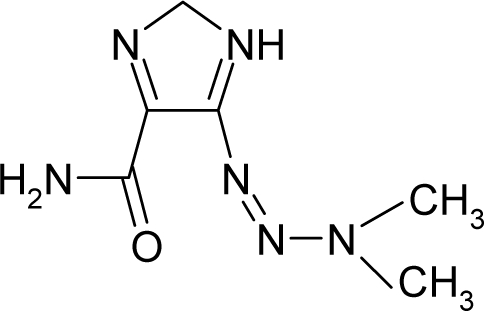	Dacarbazine C_6_H_10_N_6_O (4342-03-4)	0.71 *6.15*	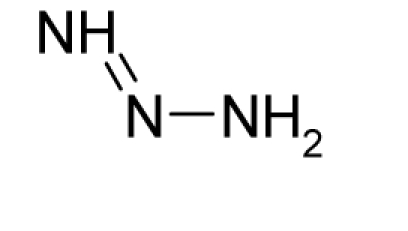	−0.92 *0.48*	17.95 *4.18*	−49126.58594 *−12249.66113*	4.9880568 *4.2572947*	4.1820822 *5.06465235*
15.	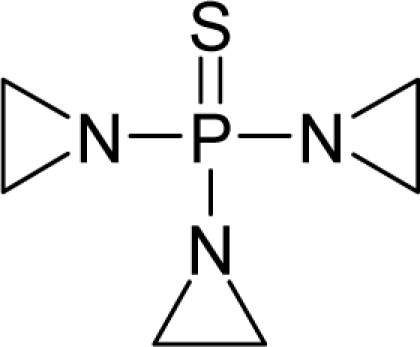	Thiotepa (Tris(aziridinyl)-phosphine sulfide) C_6_H_12_N_3_PS (52-24-4)	0.164 *6.789*	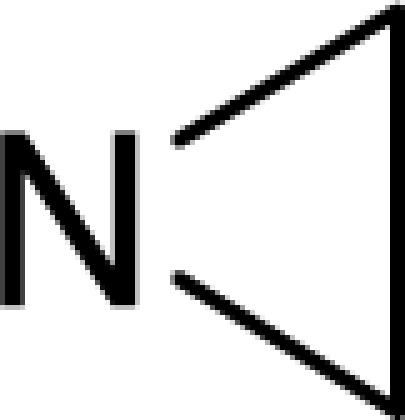	0.54 *−0.38*	17.63 *5.02*	−38905.46484 *−*1095*6.04395*	5.2831755 *3.5910075*	3.8071835 *6.3290665*
16.	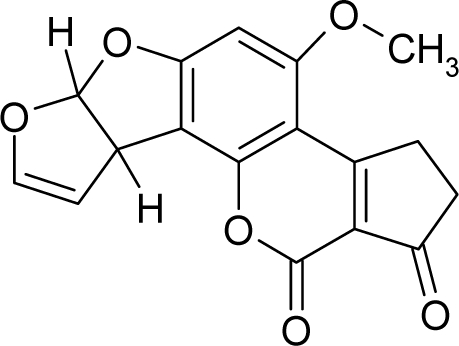	Aflatoxin-B1 C_17_H_12_O_6_ (1162-65-8)	0.0032 *8.49*	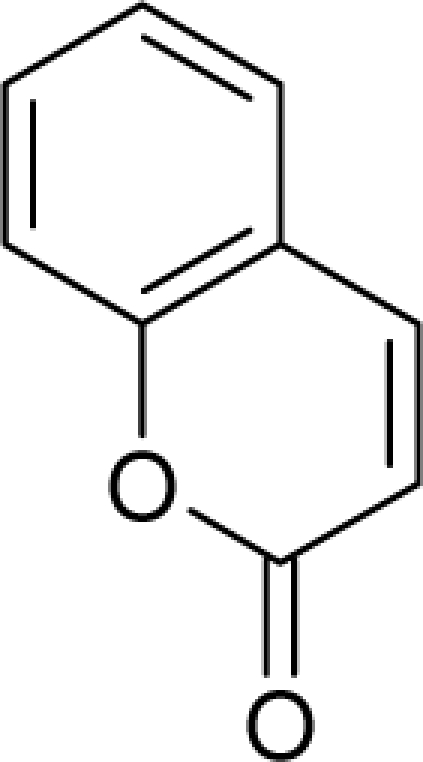	0.99 *1.82*	29.86 *15.7*	−91307.82331 *−40247.55469*	5.3273625 *5.2410253*	3.9567405 *4.2472247*
17.	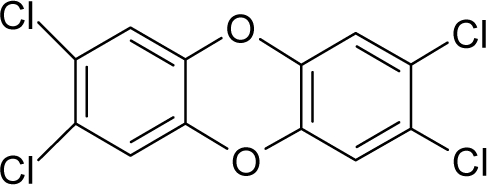	2,3,7,8-Tetrachlorodibenzo-p-dioxin C_12_H_4_Cl_4_O2 (1746-01-6)	0.0000457 *10.34*	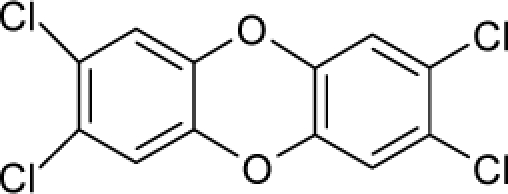	4.93 *4.93*	28.31 *28.31*	−76933.75 *−76933.75*	4.7914412 *4.7914412*	4.0075488 *4.0075488*
18.	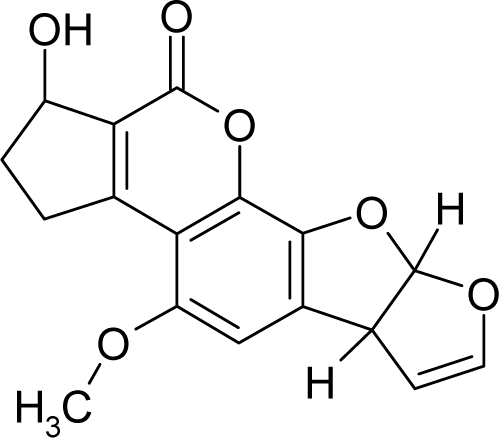	Aflatoxicol C_17_H_14_O6 (29611-03-8)	0.00247 *8.61*	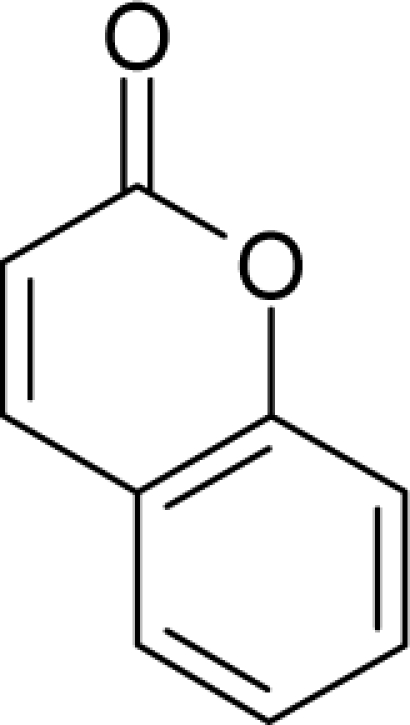	0.46 *1.82*	30.41 *15.7*	−91979.58594 *−40247.55469*	5.140259 *5.2410253*	3.945276 *4.2472247*
19.	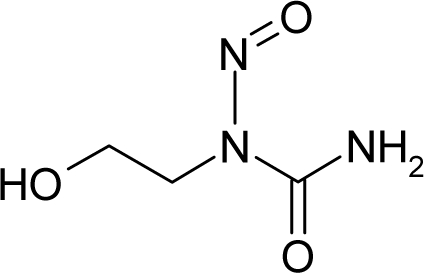	1-(2-Hydroxyethyl)-1-nitrosourea C_3_H_7_N_3_O_3_ (13743-07-2)	0.244 *6.61*	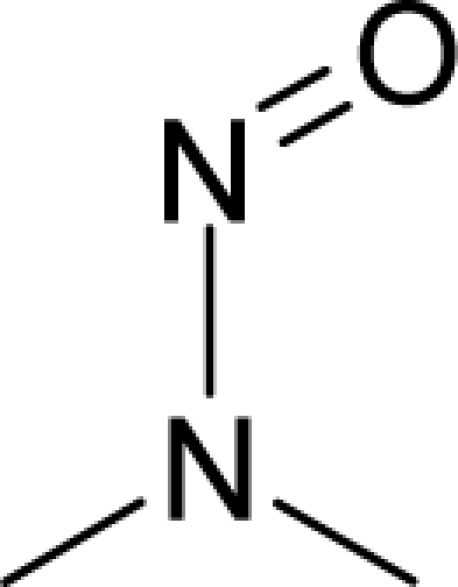	−0.95 *0.37*	10.92 *2.55*	−42184.19141 *−14202.18945*	5.42904375 *5.8565512*	5.08170625 *6.2971588*
20.	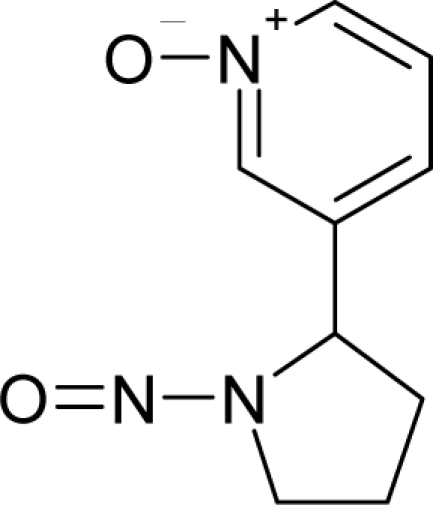	N'-Nitrosonornicotine-1-N-oxide C_9_H_11_N_3_O_2_ (78246-24-9)	0.876 *6.06*	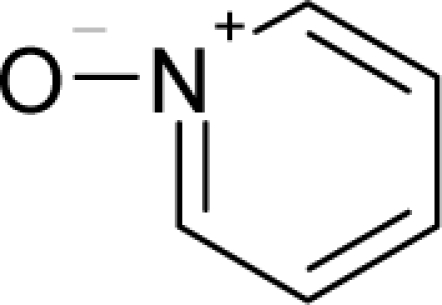	0.25 *0.12*	19.48 *10.35*	−53174.95313 *−25900.39453*	5.04527 *4.9295405*	4.273811 *4.3386305*
21.	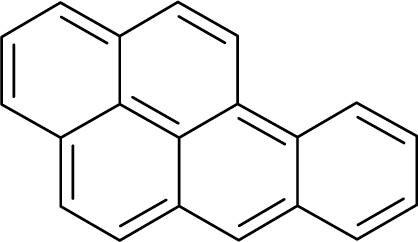	Benzo(a)pyrene C_20_H_12_ (50-32-8)	0.956 *6.02*	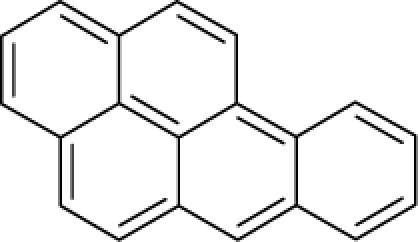	5.37 *5.37*	36.04 *36.04*	−58881.02734 *−58881.02734*	4.631374 *4.631374*	3.410258 *3.410258*
22.	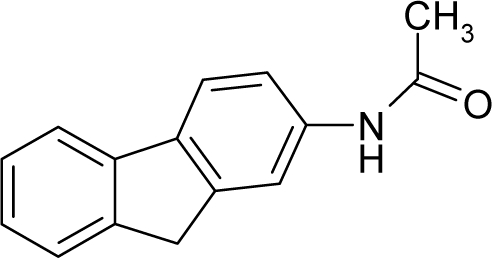	2-Acetylaminofluorene C_15_H_13_NO (53-96-3)	1.22 *5.91*	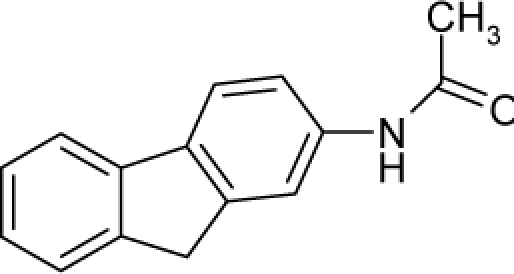	2.61 *2.61*	26.26 *26.26*	−56110.60547 *−56110.60547*	4.38615285 *4.38615285*	4.02819215 *4.02819215*
23.	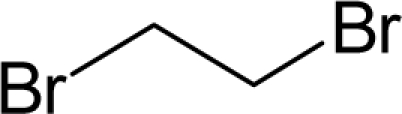	1,2-Dibromoethane C_2_H_4_Br_2_ (106-93-4)	1.52 *5.82*	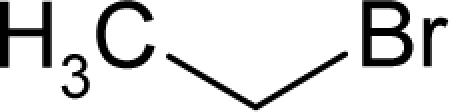	1.71 *1.29*	9.7 *7.07*	−28203.0625 *−15407.94336*	6.1527065 *5.5320367*	5.0695035 *5.37857335*
24.	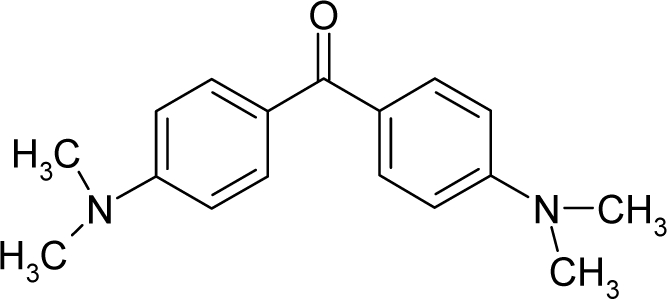	Michler's ketone C_17_H_20_N_2_O (90-94-8)	5.64 *5.25*	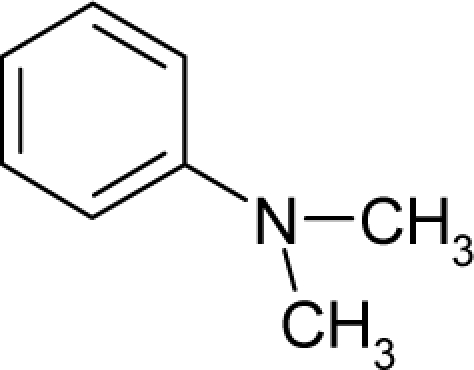	3.8 *2.31*	19.85 *15.46*	−67801.28125 *−*2950*0.11719*	4.3453716 *3.6634669*	4.1924714 *4.2943161*
25.	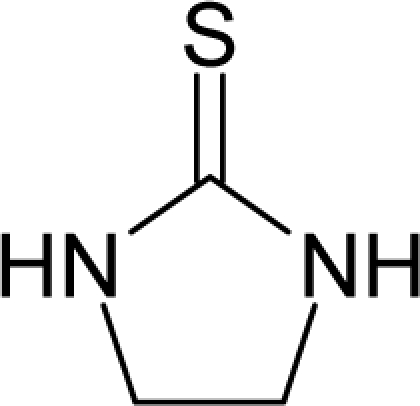	Ethylene thiourea (ETU) C_3_H_6_N_2_S (96-45-7)	8.13 *5.09*	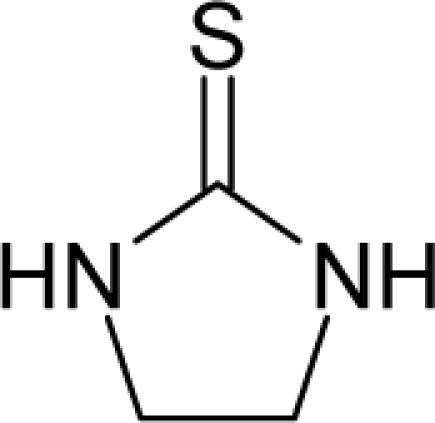	0.33 *0.33*	11.45 *11.45*	−22095.42578 *−2*2095*.42578*	4.40057075 *4.40057075*	4.20081425 *4.20081425*
26.	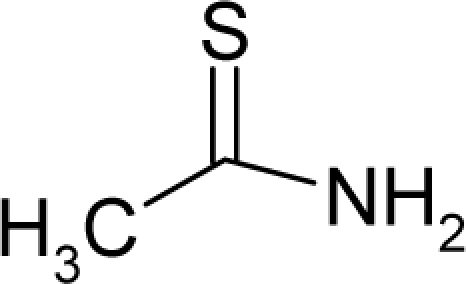	Thioacetamide C_2_H_5_NS (62-55-5)	11.5 *4.94*	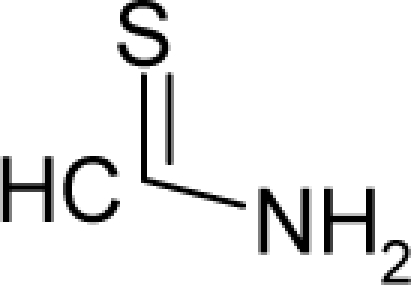	−0.21 *−0.42*	9.04 7.21	−15263.96289 *−11813.05762*	4.72959049 *4.7550568*	3.99513951 *4.0219202*
27.	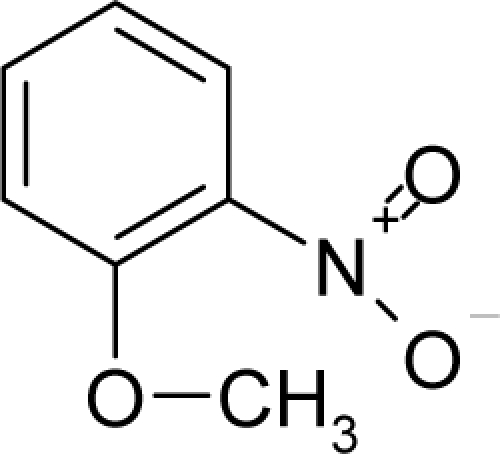	o-Nitroanisole C_7_H_7_NO_3_ (91-23-6)	15.6 *4.81*	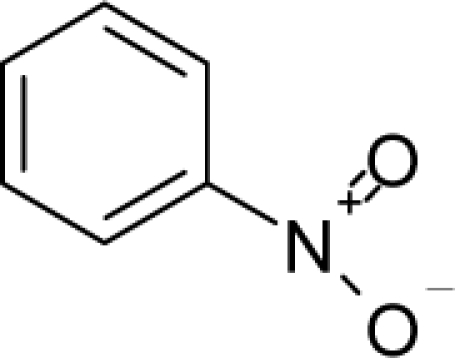	−0.18 *0.07*	14.75 *12.28*	−45613.03906 *−35381.23828*	5.5631575 *5.8686355*	4.3657605 *4.7339745*
28.	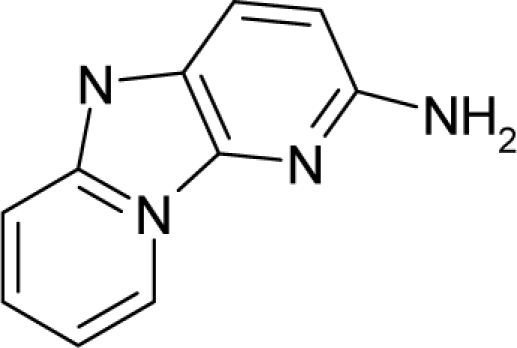	2-Aminodipyrido[1,2-a:3`,2`-d]imidazole (Glu-P-2) C_10_H_8_N_4_ (67730-10-3)	42.3 *4.37*	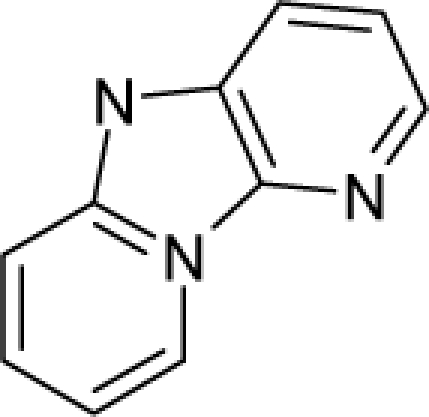	2.35 *2.9*	20.73 *19.38*	−45103.06641 *−40998.30859*	4.5267029 *4.7452532*	3.7506371 *3.87575785*
29.	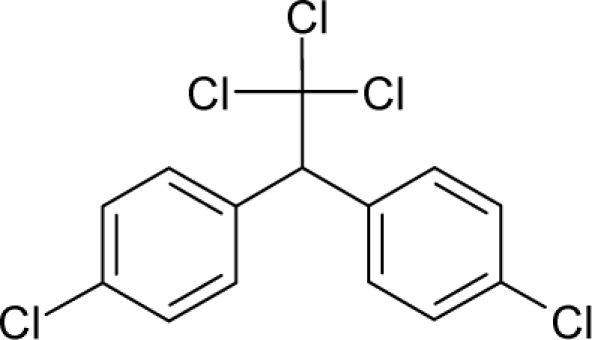	Dichlorodiphenyltrichloroet hane (DDT) C_14_H_9_Cl_5_ (50-29-3)	84.7 *4.07*	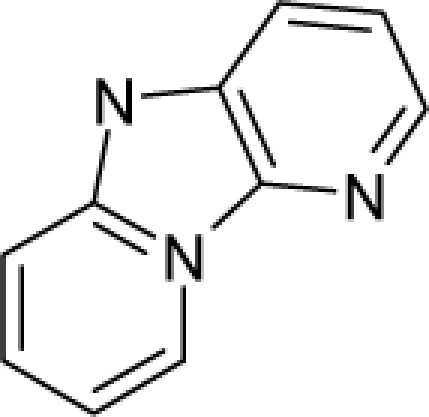	6.39 *4.92*	33.4 *25.23*	−77956.60156 *−52871.28516*	4.95182645 *5.0230205*	4.50488155 *3.2895935*
30.	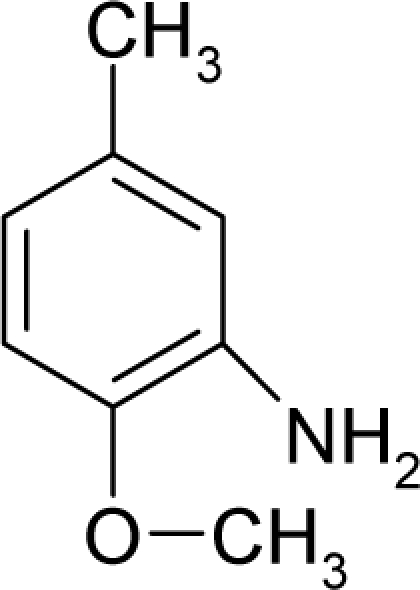	p-Cresidine C_8_H_11_NO (120-71-8)	98 *4.01*	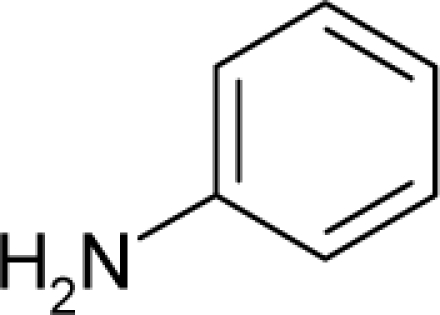	1.48 *1.26*	16.09 *11.79*	−36280.75391 *−22612.99212*	3.9300665 *3.7259962*	4.3473585 *4.3413348*
31.	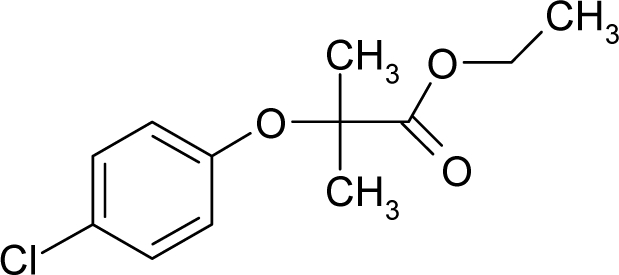	Ethyl 2-(4-chlorophenoxy)-2-methylpropionate (Clofibrate) C_12_H_15_Cl O3 (637-07-0)	169 *3.77*	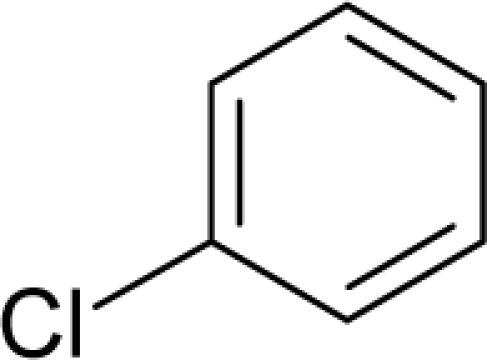	2.97 *2.56*	24.73 *12.36*	−65740.6875 *−25464.87109*	4.49111609 *4.6624658*	4.53578491 *4.72527225*
32.	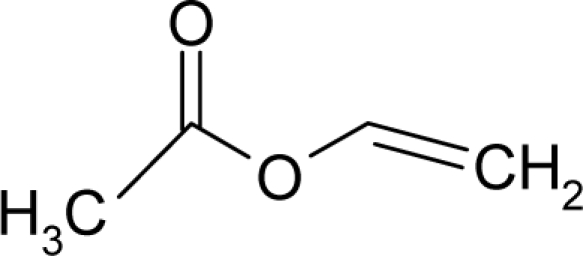	Vinyl acetate C_4_H_6_O_2_ (108-05-4)	341 *3.47*	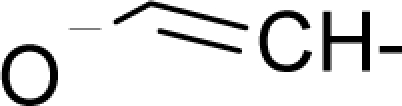	−0.01 *1.28*	8.65 *3.98*	−26598.12305 *−1*2920*.42871*	4.6849081 *4.5472153*	5.2657279 *4.91445575*
33.	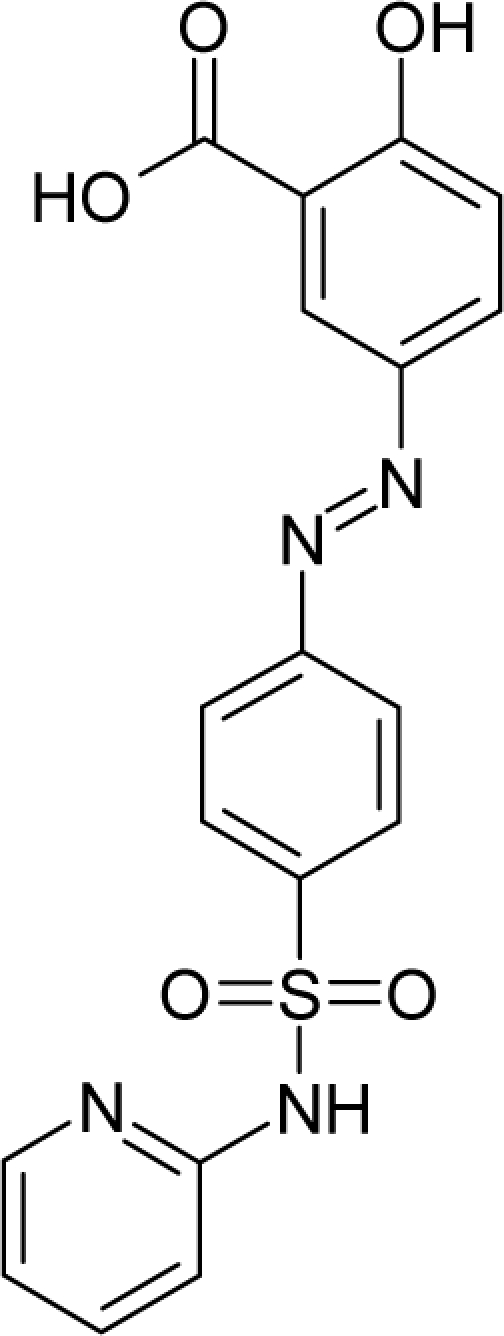	Salicylazosulfapyridine C_18_H_14_N_4_O_5_S (599-79-1)	1590 *2.799*	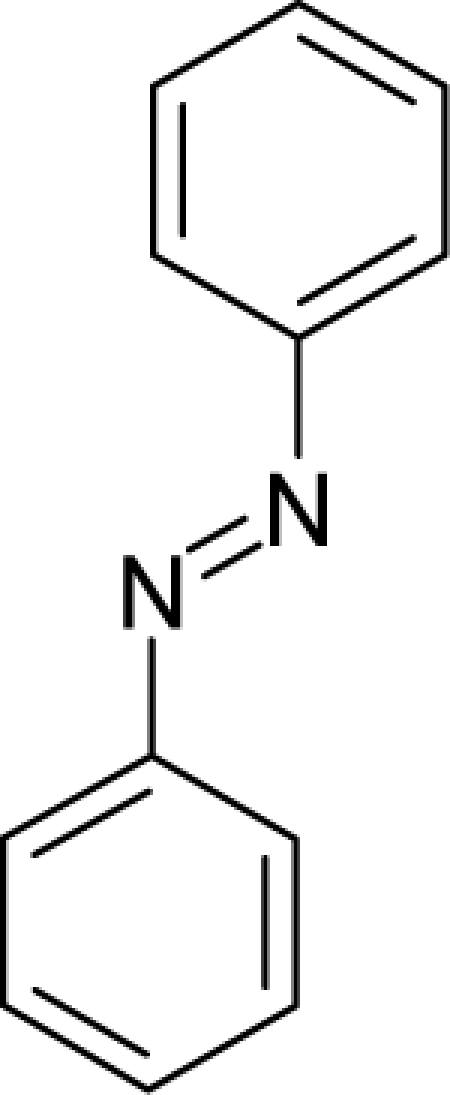	4.54 *4.35*	36.79 *22.15*	−107222.1719 *−43772.44922*	5.209331 *5.0515378*	3.898064 *4.2222632*

**Table 2.** Molecules from the quasi-Gaussian test set (Figure 2), withthe activities and structural parameters as in Table 1.

**No.**	**Full Molecule:** **Chemical Structure**	**Full Molecule:** **Name** **Formula** **(CASRN)**	**Toxicity:** **TD_50_** **Activity:** ***A = Log[1/TD****_50_****]***	**Structural Alert (SA)**	**LogP:** **Full Molecules** ***Structural Alert***	**POL [Ǻ^3^]:** **Full Molecules** ***Structural Alert***	**Etot [kcal/mol]:** **Full Molecules** ***Structural Alert***	**χ[eV]:** **Full Molecules** ***Structural Alert***	**η[eV]:** **Full Molecules** ***Structural Alert***
34.	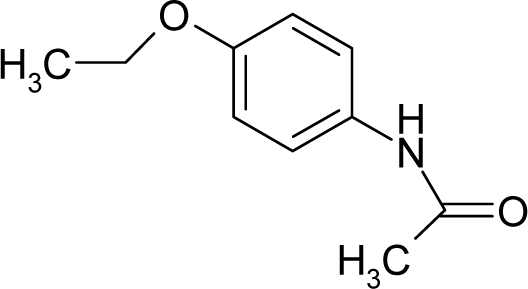	Phenacetin C_10_H_13_NO_2_ (62-44-2)	1250 *2.90*	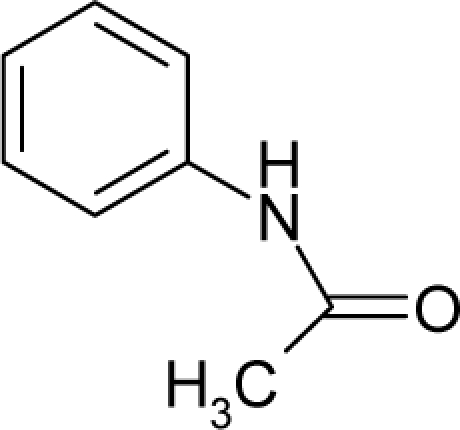	0.99 *−0.03*	19.85 *15.73*	−49230.08203 *−36279.96484*	4.063315 *4.1985829*	4.307675 *4.3648181*
35.	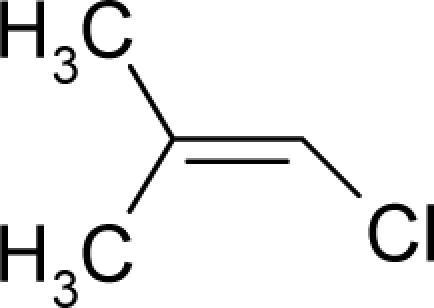	Dimethylvinyl chloride (DMVC) C_4_H_7_Cl (513-37-1)	31.8 *4.498*	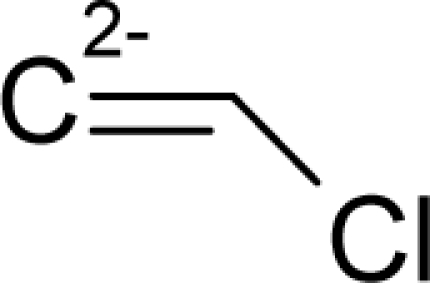	1.51 *0.45*	9.85 *4.05*	−20725.60325 *−13014.37793*	4.32596855 *5.6212095*	4.98083445 *4.2918295*
36.	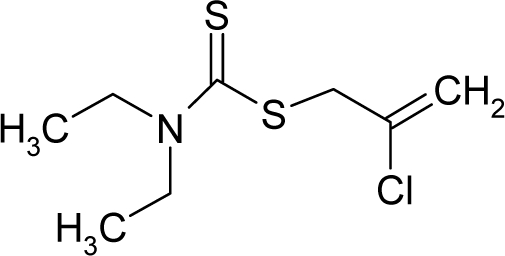	Sulfallate C_8_H_14_ClNS_2_ (95-06-7)	26.1 *4.58*	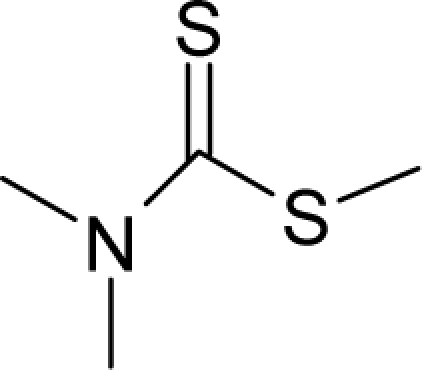	2.73 *0.62*	24.79 *10.21*	−46435.69922 *−16106.21777*	4.8447835 *5.093712*	3.8753115 *3.905288*
37.	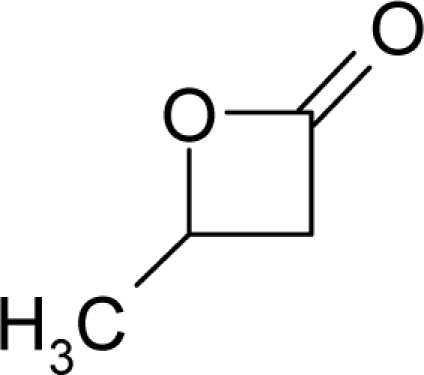	beta-Butyrolactone C_4_H_6_O_2_ (3068-88-0)	13.8 *4.86*	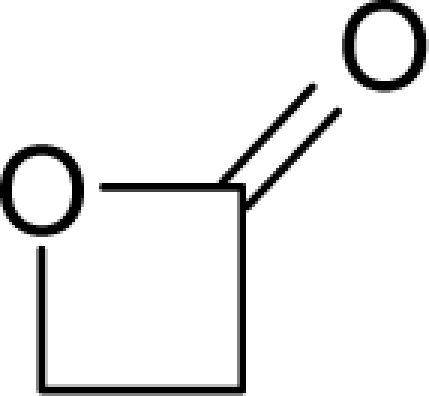	0.17 *−0.25*	8.06 *6.23*	−26599.55273 *−23148.73047*	5.1344426 *5.*2020*294*	6.0826774 *6.0841706*
38.	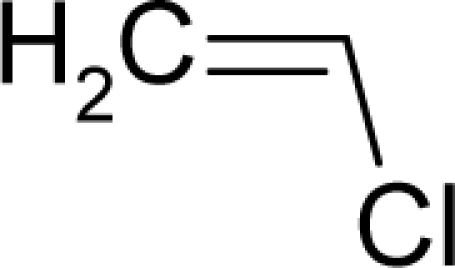	Vinyl Chloride C_2_H_3_Cl (75-01-4)	6.11 *5.21*	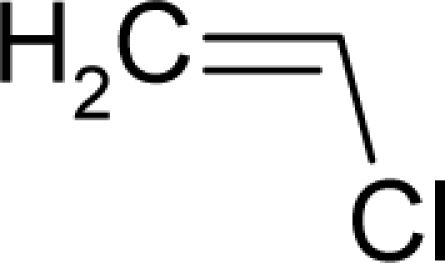	1.01 *1.01*	6.18 6.18	−13820.70898 *−13820.70898*	4.56666095 *4.56666095*	5.27117005 *5.27117005*
39.	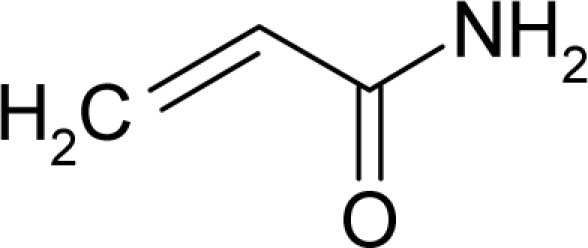	Acrylamide C_3_H_5_NO (79-06-1)	3.75 *5.43*	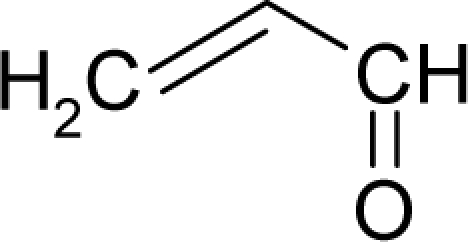	−0.28 *0.17*	7.52 *6.17*	−20478.92578 *−16372.65625*	4.77457395 *5.4404992*	4.91861805 *5.25305085*
40.	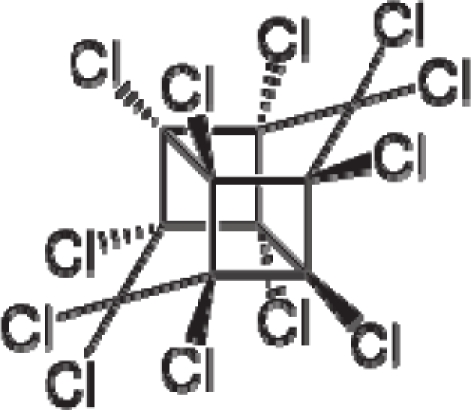	Mirex C_10_Cl_12_ (2385-85-5)	1.77 *5.75*	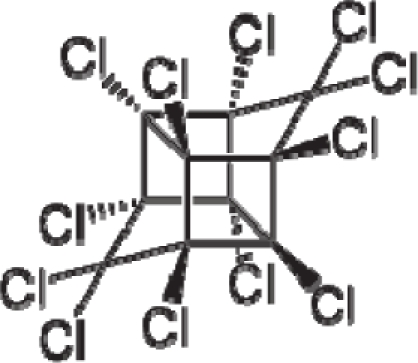	6.41 *6.41*	38.39 *38.39*	−114919.4688 *−114919.4688*	5.27780275 *5.2778028*	5.22349725 *5.22349725*
41.	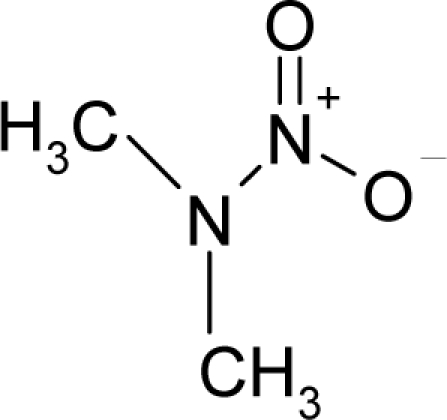	Dimethylnitramine C_2_H_6_N_2_O_2_ (4164-28-7)	0.547 *6.26*	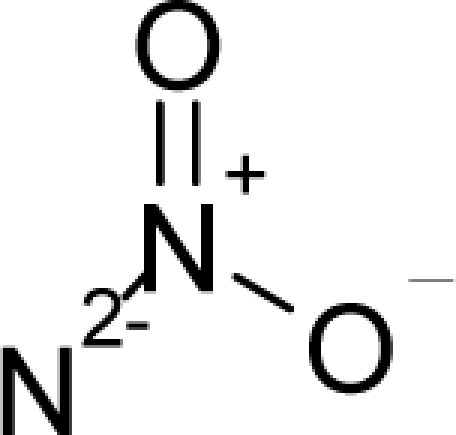	0.97 *1.32*	7.64 *3.18*	−28551.91406 *−20856.80078*	5.288693895 *7.5671675*	5.374516105 *4.9321725*
42.	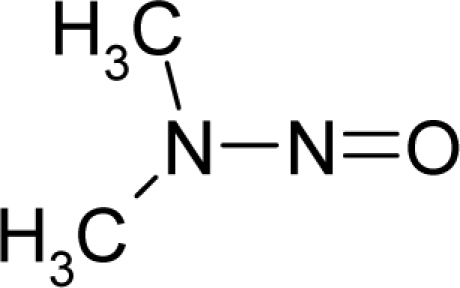	N-Nitrosodimethylami ne C_2_H_6_N_2_O (62-75-9)	0.0959 *7.02*	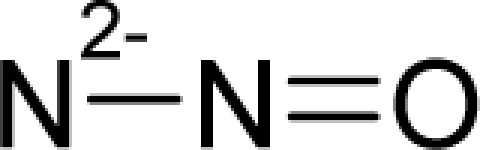	0.01 *0.37*	7.01 *2.55*	−21802.08203 *−14202.18945*	4.6046239 *5.8565512*	5.1639551 *6.2971588*
43.	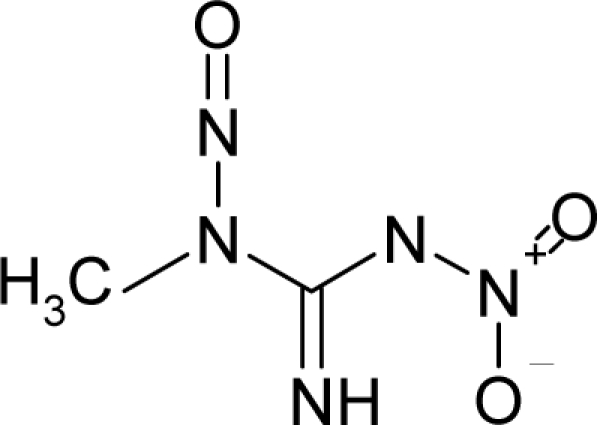	N-Methyl-N`-nitro-N-nitrosoguanidine (1-Methyl-3-nitro-1-nitroso-guanidine) C_2_H_5_N_5_O_3_ (70-25-7)	0.803 *6.1*	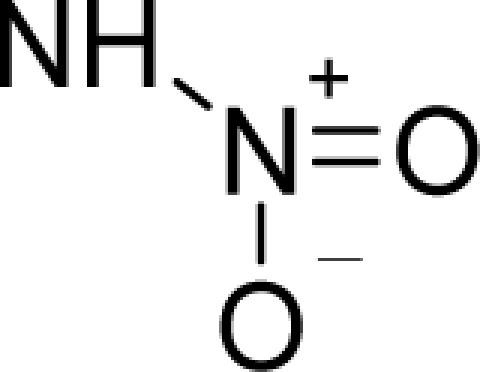	1.5 *0.84*	11.13 *3.97*	−46112.81641 *−21661.2832*	5.475207 *5.8694368*	4.654173 *5.785463245*
44.	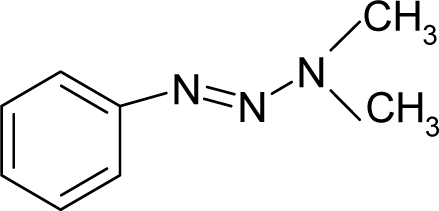	1-Phenyl-3,3-dimethyltriazene C_8_H_11_N_3_ (7227-91-0)	2.31 *5.64*	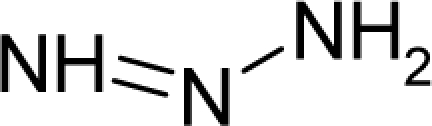	2.53 0.48	17.51 *4.18*	−36944.65625 *−12249.66113*	4.65555575 *4.2572947*	4.28693125 *5.06465235*
45.	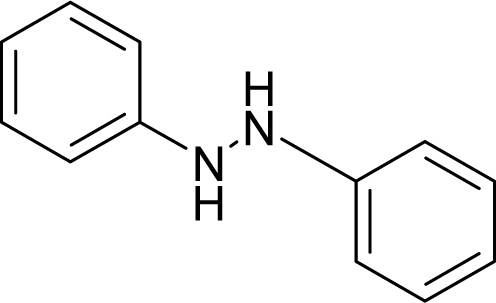	Hydrazobenzene C_12_H_12_N_2_ (122-66-7)	5.59 *5.25*	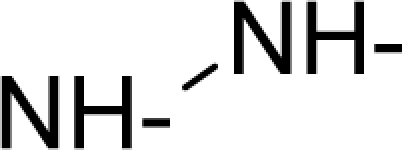	3.4 *−0.65*	22.8 *2.83*	−44481.07422 *−8164.909668*	3.65518885 *4.5593574*	3.99645815 *5.0568536*
46.	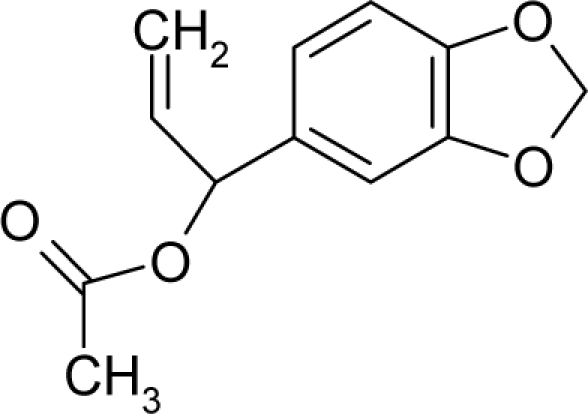	1'-Acetoxysafrole C_12_H_12_O_4_ (34627-78-6)	25 *4.6*	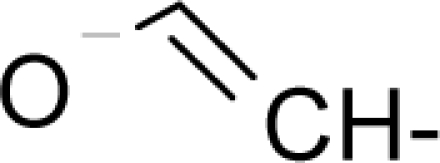	−0.11 *1.28*	22.47 *3.98*	−64108.48047 *−12920.42871*	4.516422835 *4.5472153*	4.517086165 *4.91445575*
47.	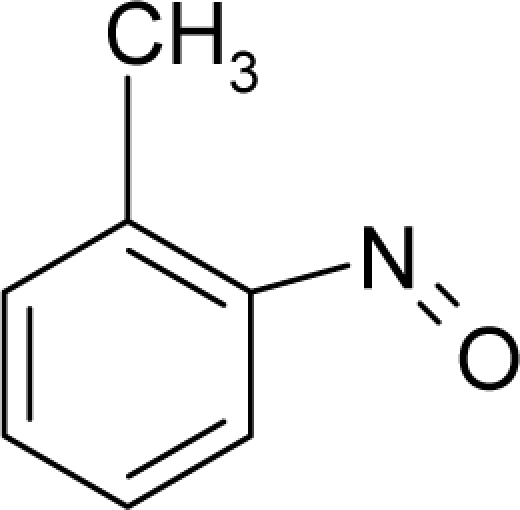	o-Nitrosotoluene C_7_H_7_NO (611-23-4)	50.7 *4.29*	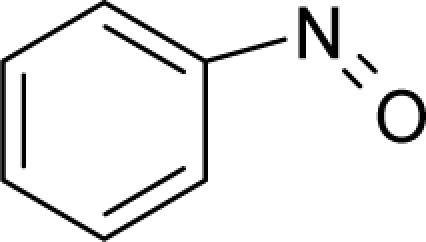	2.29 *1.82*	13.48 *11.65*	−32074.53516 *−28624.52734*	5.20234765 *5.2781928*	4.40152935 *4.43740625*
48.	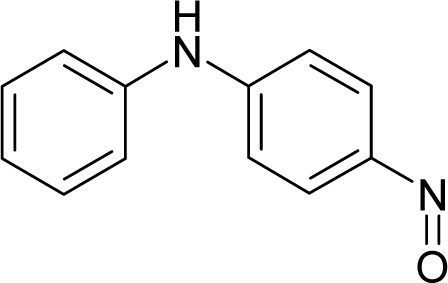	p-Nitrosodiphenyl amine C_12_H_10_N_2_O (156-10-5)	201 *3.7*	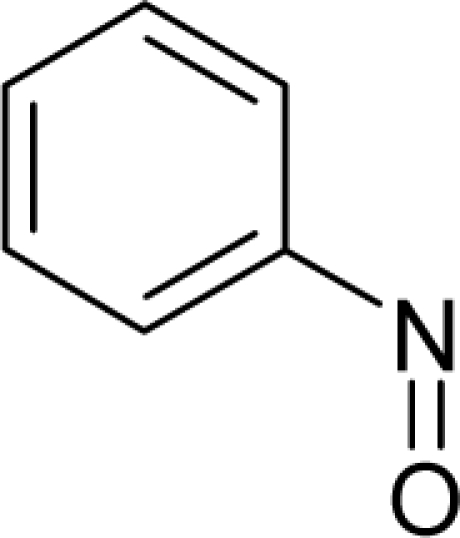	3.07 *1.82*	22.66 *11.65*	−50526.36328 *−28624.52734*	4.57337225 *5.2781928*	3.74357475 *4.43740625*
49.	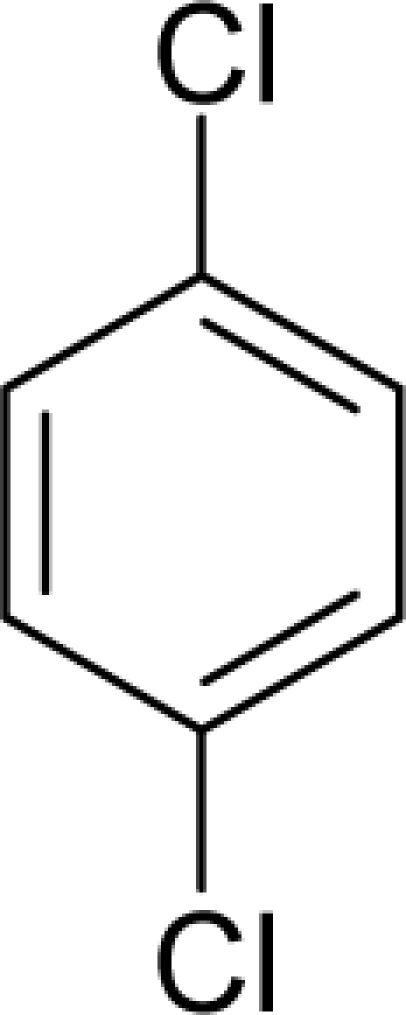	1,4-Dichlorobenzene (p-dichlorobenzene) C_6_H_4_Cl_2_ (106-46-7)	644 *3.19*	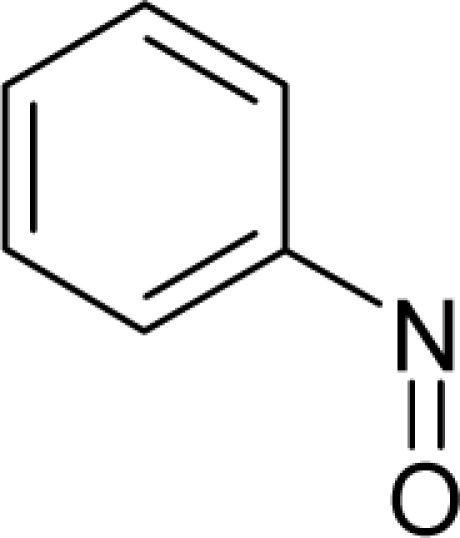	3.08 *3.08*	14.29 *14.29*	−32415.54297 *−32415.54297*	4.73892295 *4.73892295*	4.49613405 *4.49613405*

**Table 3.** QSAR models for the activities of the trial molecules with the physicochemical parameters of the SAs in Table 1.

**Variable**	**QSAR Model**	**Pred. Activity**	**R**
*χ^SA^*	5.62 − 0.07*χ^SA^*	$A_{\chi }^{\mathrm{SA}}$	0.026
*η^SA^*	6.016 − 0.149*η^SA^*	$A_{\eta }^{\mathrm{SA}}$	0.086
*POL^SA^*	4.677 + 0.05*POL^SA^*	$A_{\mathrm{POL}}^{\mathrm{SA}}$	0.27
*LogP^SA^*	5.066 + 0.159*LogP^SA^*	$A_{\mathrm{LogP}}^{\mathrm{SA}}$	0.16
$E_{\mathrm{tot}}^{\mathrm{SA}}$	4.402 − 0.00031 $E_{\mathrm{tot}}^{\mathrm{SA}}$	$A_{E\mathrm{tot}}^{\mathrm{SA}}$	0.35

*χ^SA^*, *η^SA^*	6.51 − 0.09*χ^SA^* − 0.156*η^SA^*	$A_{\chi ,\eta }^{\mathrm{SA}}$	0.093
*χ^SA^*, *POL^SA^*	5.1 − 0.08*χ^SA^* + 0.5*POL^SA^*	$A_{\chi ,\mathrm{POL}}^{\mathrm{SA}}$	0.27
*χ^SA^*, *LogP^SA^*	5.54 − 0.1*χ^SA^* + 0.16*LogP^SA^*	$A_{\chi ,\mathrm{LogP}}^{\mathrm{SA}}$	0.16
*χ^SA^*, $E_{\mathrm{tot}}^{\mathrm{SA}}$	5.58 − 0.255*χ^SA^* − 0.000032 $E_{\mathrm{tot}}^{\mathrm{SA}}$	$A_{\chi ,E\mathrm{tot}}^{\mathrm{SA}}$	0.371
*η^SA^*, *POL^SA^*	2.43 + 0.38*η^SA^* + 0.08*POL^SA^*	$A_{\eta ,\mathrm{POL}}^{\mathrm{SA}}$	0.316
*η^SA^*, *LogP^SA^*	4.75 + 0.05*η^SA^* + 0.179*LogP^SA^*	$A_{\eta ,\mathrm{LogP}}^{\mathrm{SA}}$	0.167
*η^SA^*, $E_{\mathrm{tot}}^{\mathrm{SA}}$	2.6 + 0.314*η^SA^* − 0.00004 $E_{\mathrm{tot}}^{\mathrm{SA}}$	$A_{\eta ,E\mathrm{tot}}^{\mathrm{SA}}$	0.38
*POL^SA^*, *LogP^SA^*	4.36 + 0.12*POL^SA^* − 0.44*LogP^SA^*	$A_{\mathrm{POL},\mathrm{LogP}}^{\mathrm{SA}}$	0.337
*POL^SA^*, $E_{\mathrm{tot}}^{\mathrm{SA}}$	4.38 − 0.0625*POL^SA^* − 0.00005 $E_{\mathrm{tot}}^{\mathrm{SA}}$	$A_{\mathrm{POL}.E\mathrm{tot}}^{\mathrm{SA}}$	0.382
$E_{\mathrm{tot}}^{\mathrm{SA}}$, *LogP^SA^*	4.09 − 0.412*LogP^SA^* − 0.00006 $E_{\mathrm{tot}}^{\mathrm{SA}}$	$A_{E\mathrm{tot},\mathrm{LogP}}^{\mathrm{SA}}$	0.4305

*χ^SA^*, *η^SA^*, *POL^SA^*	2.62 − 0.035*χ^SA^* + 0.382*η^SA^* + 0.08*POL^SA^*	$A_{\chi ,\eta ,\mathrm{POL}}^{\mathrm{SA}}$	0.31
*χ^SA^*, *η^SA^*, *LogP^SA^*	5.25 − 0.095*χ^SA^* + 0.049*η^SA^* + 0.179*LogP^SA^*	$A_{\chi ,\eta ,\mathrm{LogP}}^{\mathrm{SA}}$	0.17
*χ^SA^*, *η^SA^*, $E_{\mathrm{tot}}^{\mathrm{SA}}$	3.78 − 0.254*χ^SA^* + 0.3141*η^SA^* − 0.00004 $E_{\mathrm{tot}}^{\mathrm{SA}}$	$A_{\chi ,\eta ,E\mathrm{tot}}^{\mathrm{SA}}$	0.399
*χ^SA^*, *POL^SA^*, *LogP^SA^*	4.52 − 0.0325*χ^SA^* + 0.12*POL^SA^* − 0.439*LogP^SA^*	$A_{\chi ,\mathrm{POL},\mathrm{LogP}}^{\mathrm{SA}}$	0.337
*χ^SA^*, *POL^SA^*, $E_{\mathrm{tot}}^{\mathrm{SA}}$	6.46 − 0.45*χ^SA^* − 0.092*POL^SA^* − 0.0007 $E_{\mathrm{tot}}^{\mathrm{SA}}$	$A_{\chi ,\mathrm{POL},E\mathrm{tot}}^{\mathrm{SA}}$	0.4141
*χ^SA^*, *LogP^SA^*, $E_{\mathrm{tot}}^{\mathrm{SA}}$	5.75 − 0.36*χ^SA^* − 0.45*LogP^SA^* − 0.00006 $E_{\mathrm{tot}}^{\mathrm{SA}}$	$A_{\chi ,\mathrm{LogP},E\mathrm{tot}}^{\mathrm{SA}}$	0.451
*η^SA^*, *POL^SA^*, *LogP^SA^*	2.05 + 0.39*η^SA^* + 0.16*POL^SA^* − 0.449*LogP^SA^*	$A_{\eta ,\mathrm{POL},\mathrm{LogP}}^{\mathrm{SA}}$	0.373
*η^SA^*, *POL^SA^*, $E_{\mathrm{tot}}^{\mathrm{SA}}$	3.09 + 0.22*η^SA^* − 0.034*POL^SA^* − 0.00005 $E_{\mathrm{tot}}^{\mathrm{SA}}$	$A_{\eta ,\mathrm{POL},E\mathrm{tot}}^{\mathrm{SA}}$	0.392
*η^SA^*, *LogP^SA^*, $E_{\mathrm{tot}}^{\mathrm{SA}}$	3.23 + 0.156*η^SA^* − 0.368*LogP^SA^* − 0.0006 $E_{\mathrm{tot}}^{\mathrm{SA}}$	$A_{\eta ,\mathrm{LogP},E\mathrm{tot}}^{\mathrm{SA}}$	0.436
*POL^SA^*, *LogP^SA^*, $E_{\mathrm{tot}}^{\mathrm{SA}}$	4.07 + 0.015*POL^SA^* − 0.45*LogP^SA^* − 0.000057 $E_{\mathrm{tot}}^{\mathrm{SA}}$	$A_{\mathrm{POL},\mathrm{LogP},E\mathrm{tot}}^{\mathrm{SA}}$	0.4312

**Table 4.** Multi-linear QSAR models for the trial molecular activities with the full molecular (M) physicochemical parameters of Table 1 and the corresponding activities of the structural alerts (A^SA^ or ASA) from Table 3.

**Variable**	**QSAR Model**	**Pred. Activity**	**R**
*χ^M^*	2.18 + 0.63*χ^M^*	$A_{\chi }^{M}$	0.187
*χ^M^*, $A_{\chi }^{\mathrm{SA}}$	− 65.66 + 12.1 $A_{\chi }^{\mathrm{SA}}$ + 1.41*χ^M^*	$A_{\chi }^{M\wedge \mathrm{ASA}}$	0.295
*η^M^*	7.13 − 0.405 *η^M^*	$A_{\eta }^{M}$	0.192
*η^M^*, $A_{\eta }^{\mathrm{SA}}$	11.38 − 0731 $A_{\eta }^{\mathrm{SA}}$ − 0.49 *η^M^*	$A_{\eta }^{M\wedge \mathrm{ASA}}$	0.198
*POL^M^*	4.71 + 0.029*POL^M^*	$A_{\mathrm{POL}}^{M}$	0.18
*POL^M^*, $A_{\mathrm{POL}}^{\mathrm{SA}}$	− 0.438 + 1.1 $A_{\mathrm{POL}}^{\mathrm{SA}}$ − 0.006*POL^M^*	$A_{\mathrm{POL}}^{M\wedge \mathrm{ASA}}$	0.275
*LogP^M^*	5.29 − 0.0007*LogP^M^*	$A_{\mathrm{POL}}^{M}$	0.009
*LogP^M^*, $A_{\mathrm{LogP}}^{\mathrm{SA}}$	− 13.64 + 3.71 $A_{\mathrm{LogP}}^{\mathrm{SA}}$ − 0.421*LogP^M^*	$A_{\mathrm{POL}}^{M\wedge \mathrm{ASA}}$	0.325
$E_{\mathrm{tot}}^{M}$	4.67 − 0.00001 $E_{\mathrm{TOT}}^{M}$	$A_{E\mathrm{tot}}^{M}$	0.203
$E_{\mathrm{tot}}^{M}$, $A_{E\mathrm{tot}}^{\mathrm{SA}}$	− 0.05 + 1.01 $A_{E\mathrm{tot}}^{\mathrm{SA}}$ + 0.000001 $E_{\mathrm{tot}}^{M}$	$A_{E\mathrm{tot}}^{M\wedge \mathrm{ASA}}$	0.358

*χ^M^*, *η^M^*	3.92 + 0.68*χ^M^* − 0.43*η^M^*	$A_{\chi ,\eta }^{M}$	0.278
*χ^M^*, *η^M^*, $A_{\chi ,\eta }^{\mathrm{SA}}$	0.015 + 0.73*χ^M^* − 0.35*η^M^* + 0.62 $A_{\chi ,\eta }^{\mathrm{SA}}$	$A_{\chi ,\eta }^{M\wedge \mathrm{ASA}}$	0.28
*χ^M^*, *POL^M^*	1.36 + 0.67*χ^M^* + 0.031*POL^M^*	$A_{\chi ,\mathrm{POL}}^{M}$	0.27
*χ^M^*, *POL^M^*, $A_{\chi ,\mathrm{POL}}^{\mathrm{SA}}$	− 6.009 + 0.86*χ^M^* − 0.012*POL^M^* + 1.38 $A_{\chi ,\mathrm{POL}}^{\mathrm{SA}}$	$A_{\chi ,\mathrm{POL}}^{M\wedge \mathrm{ASA}}$	0.373
*χ^M^*, *LogP^M^*	2.12 + 0.639*χ^M^* + 0.013*LogP^M^*	$A_{\chi ,\mathrm{LogP}}^{M}$	0.188
*χ^M^*, *LogP^M^*, $A_{\chi ,\mathrm{LogP}}^{\mathrm{SA}}$	− 19.46 + 0.81*χ^M^* − 0.44*LogP^M^* + 4.05 $A_{\chi ,\mathrm{LogP}}^{\mathrm{SA}}$	$A_{\chi ,\mathrm{LogP}}^{M\wedge \mathrm{ASA}}$	0.409
*χ^M^*, $E_{\mathrm{tot}}^{M}$	1.64 + 0.62*χ^M^* − 0.00001 $E_{\mathrm{tot}}^{M}$	$A_{\chi ,E\mathrm{tot}}^{M}$	0.27
*χ^M^*, $E_{\mathrm{tot}}^{M}$, $A_{\chi ,E\mathrm{tot}}^{\mathrm{SA}}$	− 4.81 + 0.83*χ^M^* + 0.00003 $E_{\mathrm{tot}}^{M}$ + 1.16 $A_{\chi ,E\mathrm{tot}}^{\mathrm{SA}}$	$A_{\chi ,E\mathrm{tot}}^{M\wedge \mathrm{ASA}}$	0.443
*η^M^*, *POL^M^*	6.18 − 0.264*η^M^* + 0.015*POL^M^*	$A_{\eta ,\mathrm{POL}}^{M}$	0.204
*η^M^*, *POL^M^*, $A_{\eta ,\mathrm{POL}}^{\mathrm{SA}}$	1.47 − 0.64*η^M^* − 0.05*POL^M^* + 1.48 $A_{\eta ,\mathrm{POL}}^{\mathrm{SA}}$	$A_{\eta ,\mathrm{POL}}^{M\wedge \mathrm{ASA}}$	0.378
*η^M^*, *LogP^M^*	7.66 − 0.49*η^M^* − 0.08*LogP^M^*	$A_{\eta ,\mathrm{LogP}}^{M}$	0.213
*η^M^*, *LogP^M^*, $A_{\eta ,\mathrm{LogP}}^{\mathrm{SA}}$	−12.17 − 0.52*η^M^* − 0.531*LogP^M^* + 3.919 $A_{\eta ,\mathrm{LogP}}^{\mathrm{SA}}$	$A_{\eta ,\mathrm{LogP}}^{M\wedge \mathrm{ASA}}$	0.403
*η^M^*, $E_{\mathrm{tot}}^{M}$	5.993 − 0.24*η^M^* − 0.000008 $E_{\mathrm{tot}}^{M}$	$A_{\eta ,E\mathrm{tot}}^{M}$	0.226
*η^M^*, $E_{\mathrm{tot}}^{M}$, $A_{\eta ,E\mathrm{tot}}^{\mathrm{SA}}$	1.585 − 0.442*η^M^* + 0.00001 $E_{\mathrm{tot}}^{M}$ + 1.182 $A_{\eta ,E\mathrm{tot}}^{\mathrm{SA}}$	$A_{\eta ,E\mathrm{tot}}^{M\wedge \mathrm{ASA}}$	0.426
*POL^M^*, *LogP^M^*	4.339 + 0.72*POL^M^* − 0.279*LogP^M^*	$A_{\mathrm{POL},\mathrm{LogP}}^{M}$	0.29
*POL^M^*, *LogP^M^*, $A_{\mathrm{POL},\mathrm{LogP}}^{\mathrm{SA}}$	− 0.81 + 0.04*POL^M^* − 0.31*LogP^M^* + 1.08 $A_{\mathrm{POL},\mathrm{LogP}}^{\mathrm{SA}}$	$A_{\mathrm{POL},\mathrm{LogP}}^{M\wedge \mathrm{ASA}}$	0.422
*POL^M^*, $E_{\mathrm{tot}}^{M}$	4.67 − 0.00097*POL^M^* − 0.00001 $E_{\mathrm{tot}}^{M}$	$A_{\mathrm{POL},E\mathrm{tot}}^{M}$	0.203
*POL^M^*, $E_{\mathrm{tot}}^{M}$, $A_{\mathrm{POL},E\mathrm{tot}}^{\mathrm{SA}}$	− 0.299 − 0.0304*POL^M^* − 0.00008 $E_{\mathrm{tot}}^{M}$ + 1.091 $A_{\mathrm{POL},E\mathrm{tot}}^{\mathrm{SA}}$	$A_{\mathrm{POL},E\mathrm{tot}}^{M\wedge \mathrm{ASA}}$	0.3907
$E_{\mathrm{tot}}^{M}$, *LogP^M^*	4.578 − 0.162*LogP^M^* − 0.000018 $E_{\mathrm{tot}}^{M}$	$A_{E\mathrm{tot},\mathrm{LogP}}^{M}$	0.26
$E_{\mathrm{tot}}^{M}$, *LogP^M^*, $A_{E\mathrm{tot},\mathrm{LogP}}^{\mathrm{SA}}$	− 0.603 − 0.2337*LogP^M^* − 0.00009 $E_{\mathrm{tot}}^{M}$ + 1.093 $A_{E\mathrm{tot},\mathrm{LogP}}^{\mathrm{SA}}$	$A_{E\mathrm{tot},\mathrm{LogP}}^{M\wedge \mathrm{ASA}}$	0.488

*χ^M^*, *η^M^*, *POL^M^*	2.89 + 0.686*χ^M^* − 0.28*η^M^* + 0.016*POL^M^*	$A_{\chi ,\eta ,\mathrm{POL}}^{M}$	0.288
*χ^M^*, *η^M^*, *POL^M^*, $A_{\chi ,\eta ,\mathrm{POL}}^{\mathrm{SA}}$	− 3.19 + 0.86*χ^M^* − 0.696*η^M^* − 0.06*POL^M^* + 1.62 $A_{\chi ,\eta ,\mathrm{POL}}^{\mathrm{SA}}$	$A_{\chi ,\eta ,\mathrm{POL}}^{M\wedge \mathrm{ASA}}$	0.452
*χ^M^*, *η^M^*, *LogP^M^*	4.458 + 0.65*χ^M^* − 0.5*η^M^* − 0.06*LogP^M^*	$A_{\chi ,\eta ,\mathrm{LogP}}^{M}$	0.287
*χ^M^*, *η^M^*, *LogP^M^*, $A_{\chi ,\eta ,\mathrm{LogP}}^{\mathrm{SA}}$	− 16.899 + 0.794*χ^M^* − 0.49*η^M^* − 0.52*LogP^M^* + 4.054 $A_{\chi ,\eta ,\mathrm{LogP}}^{\mathrm{SA}}$	$A_{\chi ,\eta ,\mathrm{LogP}}^{M\wedge \mathrm{ASA}}$	0.462
*χ^M^*, *η^M^*, $E_{\mathrm{tot}}^{M}$	3.016 + 0.65*χ^M^* − 0.293*η^M^* − 0.00007 $E_{\mathrm{tot}}^{M}$	$A_{\chi ,\eta ,E\mathrm{tot}}^{M}$	0.298
*χ^M^*, *η^M^*, $E_{\mathrm{tot}}^{M}$, $A_{\chi ,\eta ,E\mathrm{tot}}^{\mathrm{SA}}$	− 2.689 + 0.841*χ^M^* − 0.473*η^M^* + 0.00001 $E_{\mathrm{tot}}^{M}$ + 1.256 $A_{\chi ,\eta ,E\mathrm{tot}}^{\mathrm{SA}}$	$A_{\chi ,\eta ,E\mathrm{tot}}^{M\wedge \mathrm{ASA}}$	0.497
*χ^M^*, *POL^M^*, *LogP^M^*	1.481 + 0.58 *χ^M^* + 0.07*POL^M^* − 0.2512*LogP^M^*	$A_{\chi ,POL,LogP}^{M}$	0.338
*χ^M^*, *POL^M^*, *LogP^M^*, $A_{\chi ,POL,LogP}^{\mathrm{SA}}$	− 5.593 + 0.81*χ^M^* + 0.037*POL^M^* − 0.281*LogP^M^* + 1.254 $A_{\chi ,POL,LogP}^{\mathrm{SA}}$	$A_{\chi ,POL,LogP}^{M\wedge \mathrm{ASA}}$	0.483
*χ^M^*, *POL^M^*, $E_{\mathrm{tot}}^{M}$	1.503 + 0.64*χ^M^* + 0.012*POL_M_* − 0.000007 $E_{\mathrm{tot}}^{M}$	$A_{\chi ,POL,E\mathrm{tot}}^{M}$	0.276
*χ^M^*, *POL^M^*, $E_{\mathrm{tot}}^{M}$, $A_{\chi ,POL,E\mathrm{tot}}^{\mathrm{SA}}$	− 4.009 + 0.725*χ^M^* −0.008*POL^M^* + 0.00001 $E_{\mathrm{tot}}^{M}$ + 1.123 $A_{\chi ,POL,E\mathrm{tot}}^{\mathrm{SA}}$	$A_{\chi ,POL,E\mathrm{tot}}^{M\wedge \mathrm{ASA}}$	0.469
*χ^M^*, *LogP^M^*, $E_{\mathrm{tot}}^{M}$	1.961 + 0.538*χ^M^* − 0.134*LogP^M^* − 0.000017 $E_{\mathrm{tot}}^{M}$	$A_{\chi ,LogP,E\mathrm{tot}}^{M}$	0.304
*χ^M^*, *LogP^M^*, $E_{\mathrm{tot}}^{M}$, $A_{\chi ,LogP,E\mathrm{tot}}^{\mathrm{SA}}$	− 4.251 + 0.68*χ^M^* − 0.219*LogP^M^* − 0.00007 $E_{\mathrm{tot}}^{M}$ + 1.166 $A_{\chi ,LogP,E\mathrm{tot}}^{\mathrm{SA}}$	$A_{\chi ,LogP,E\mathrm{tot}}^{M\wedge \mathrm{ASA}}$	0.552
*η^M^*, *POL^M^*, *LogP^M^*	4.87 − 0.093*η^M^* + 0.066*POL^M^* − 0.269*LogP^M^*	$A_{\eta ,POL,LogP}^{M}$	0.294
*η^M^*, *POL^M^*, *LogP^M^*, $A_{\eta ,POL,LogP}^{\mathrm{SA}}$	− 0.253 − 0.153*η^M^* + 0.033*POL^M^* − 0.321*LogP^M^* + 1.156 $A_{\eta ,POL.LogP}^{\mathrm{SA}}$	$A_{\eta ,POL,LogP}^{M\wedge \mathrm{ASA}}$	0.466
*η^M^*, *POL^M^*, $E_{\mathrm{tot}}^{M}$	6.84 − 0.39 *η^M^* − 0.368*POL^M^* − 0.00018 $E_{\mathrm{tot}}^{M}$	$A_{\eta ,POL,E\mathrm{tot}}^{M}$	0.239
*η^M^*, *POL^M^*, $E_{\mathrm{tot}}^{M}$, $A_{\eta ,POL,E\mathrm{tot}}^{\mathrm{SA}}$	3.415 − 1.08*η^M^* − 0.13*POL^M^* − 0.00002 $E_{\mathrm{tot}}^{M}$ + 1.598 $A_{\eta ,POL,E\mathrm{tot}}^{\mathrm{SA}}$	$A_{\eta ,POL,E\mathrm{tot}}^{M\wedge \mathrm{ASA}}$	0.507
*η^M^*, *LogP^M^*, $E_{\mathrm{tot}}^{M}$	6.176 − 0.303*η^M^* − 0.177*LogP^M^* − 0.000015 $E_{\mathrm{tot}}^{M}$	$A_{\eta ,LogP,E\mathrm{tot}}^{M}$	0.287
*η^M^*, *LogP^M^*, $E_{\mathrm{tot}}^{M}$, $A_{\eta ,LogP,E\mathrm{tot}}^{\mathrm{SA}}$	1.433 − 0.449*η^M^* − 0.261*LogP^M^* − 0.00003 $E_{\mathrm{tot}}^{M}$ + 1.172 $A_{\eta ,LogP,E\mathrm{tot}}^{\mathrm{SA}}$	$A_{\eta ,LogP,E\mathrm{tot}}^{M\wedge \mathrm{ASA}}$	0.525
*POL^M^*, *LogP^M^*, $E_{\mathrm{tot}}^{M}$	4.341 + 0.06*POL^M^* − 0.273*LogP^M^* − 0.000002 $E_{\mathrm{tot}}^{M}$	$A_{POL,LogP,E\mathrm{tot}}^{M}$	0.298
*POL^M^*, *LogP^M^*, $E_{\mathrm{tot}}^{M}$, $A_{POL,LogP,E\mathrm{tot}}^{\mathrm{SA}}$	− 0.598 + 0.031*POL^M^* − 0.288*LogP^M^* − 0.000002 $E_{\mathrm{tot}}^{M}$ + 1.065 $A_{POL,LogP,E\mathrm{tot}}^{\mathrm{SA}}$	$A_{POL,LogP,E\mathrm{tot}}^{M\wedge \mathrm{ASA}}$	0.495

**Table 5.** Residual-QSARs for the structural alert models of Table 3.

**Variable**	**QSAR Model**	**Pred. Activity**	**R**
${\mathrm{RA}}_{\chi }^{\mathrm{SA}}$	5.2856 + ${\mathrm{RA}}_{\chi }^{\mathrm{SA}}$	${\mathrm{ARA}}_{\chi }^{\mathrm{SA}}$	0.999
${\mathrm{RA}}_{\eta }^{\mathrm{SA}}$	5.2856 + ${\mathrm{RA}}_{\eta }^{\mathrm{SA}}$	${\mathrm{ARA}}_{\eta }^{\mathrm{SA}}$	0.996
${\mathrm{RA}}_{POL}^{\mathrm{SA}}$	5.2856 + ${\mathrm{RA}}_{POL}^{\mathrm{SA}}$	${\mathrm{ARA}}_{POL}^{\mathrm{SA}}$	0.961
${\mathrm{RA}}_{LogP}^{\mathrm{SA}}$	5.2856 + ${\mathrm{RA}}_{LogP}^{\mathrm{SA}}$	${\mathrm{ARA}}_{LogP}^{\mathrm{SA}}$	0.986
${\mathrm{RA}}_{E\mathrm{tot}}^{\mathrm{SA}}$	5.2856 + ${\mathrm{RA}}_{E\mathrm{tot}}^{\mathrm{SA}}$	${\mathrm{ARA}}_{E\mathrm{tot}}^{\mathrm{SA}}$	0.933

${\mathrm{RA}}_{\chi }^{\mathrm{SA}}$, ${\mathrm{RA}}_{\eta }^{\mathrm{SA}}$	5.2856 + 0.886 ${\mathrm{RA}}_{\chi }^{\mathrm{SA}}$ + 0.114 ${\mathrm{RA}}_{\eta }^{\mathrm{SA}}$	${\mathrm{ARA}}_{\chi ,\eta }^{\mathrm{SA}}$	0.999
${\mathrm{RA}}_{\chi }^{\mathrm{SA}}$, ${\mathrm{RA}}_{POL}^{\mathrm{SA}}$	5.2856 + 0.987 ${\mathrm{RA}}_{\chi }^{\mathrm{SA}}$ + 0.013 ${\mathrm{RA}}_{POL}^{\mathrm{SA}}$	${\mathrm{ARA}}_{\chi ,POL}^{\mathrm{SA}}$	0.999
${\mathrm{RA}}_{\chi }^{\mathrm{SA}}$, ${\mathrm{RA}}_{LogP}^{\mathrm{SA}}$	5.2856 + 0.963 ${\mathrm{RA}}_{\chi }^{\mathrm{SA}}$ + 0.037 ${\mathrm{RA}}_{LogP}^{\mathrm{SA}}$	${\mathrm{ARA}}_{\chi ,LogP}^{\mathrm{SA}}$	0.999
${\mathrm{RA}}_{\chi }^{\mathrm{SA}}$, ${\mathrm{RA}}_{E\mathrm{tot}}^{\mathrm{SA}}$	5.2856 + 0.98 ${\mathrm{RA}}_{\chi }^{\mathrm{SA}}$ + 0.022 ${\mathrm{RA}}_{E\mathrm{tot}}^{\mathrm{SA}}$	${\mathrm{ARA}}_{\chi ,E\mathrm{tot}}^{\mathrm{SA}}$	0.999
${\mathrm{RA}}_{\eta }^{\mathrm{SA}}$, ${\mathrm{RA}}_{POL}^{\mathrm{SA}}$	5.2856 + 1.194 ${\mathrm{RA}}_{\eta }^{\mathrm{SA}}$ − 0.206 ${\mathrm{RA}}_{POL}^{\mathrm{SA}}$	${\mathrm{ARA}}_{\eta ,POL}^{\mathrm{SA}}$	0.997
${\mathrm{RA}}_{\eta }^{\mathrm{SA}}$, ${\mathrm{RA}}_{LogP}^{\mathrm{SA}}$	5.2856 + 1.101 ${\mathrm{RA}}_{\eta }^{\mathrm{SA}}$ − 0.103 ${\mathrm{RA}}_{LogP}^{\mathrm{SA}}$	${\mathrm{ARA}}_{\eta ,LogP}^{\mathrm{SA}}$	0.996
${\mathrm{RA}}_{\eta }^{\mathrm{SA}}$, ${\mathrm{RA}}_{E\mathrm{tot}}^{\mathrm{SA}}$	5.2856 + 1.105 ${\mathrm{RA}}_{\eta }^{\mathrm{SA}}$ − 0.118 ${\mathrm{RA}}_{E\mathrm{tot}}^{\mathrm{SA}}$	${\mathrm{ARA}}_{\eta ,E\mathrm{tot}}^{\mathrm{SA}}$	0.996
${\mathrm{RA}}_{POL}^{\mathrm{SA}}$, ${\mathrm{RA}}_{LogP}^{\mathrm{SA}}$	5.2856 − 0.709 ${\mathrm{RA}}_{POL}^{\mathrm{SA}}$ + 1.684 ${\mathrm{RA}}_{LogP}^{\mathrm{SA}}$	${\mathrm{ARA}}_{POL,LogP}^{\mathrm{SA}}$	0.991
${\mathrm{RA}}_{POL}^{\mathrm{SA}}$, ${\mathrm{RA}}_{E\mathrm{tot}}^{\mathrm{SA}}$	5.2856 + 1.642 ${\mathrm{RA}}_{POL}^{\mathrm{SA}}$ − 0.670 ${\mathrm{RA}}_{E\mathrm{tot}}^{\mathrm{SA}}$	${\mathrm{ARA}}_{POL,E\mathrm{tot}}^{\mathrm{SA}}$	0.966
${\mathrm{RA}}_{LogP}^{\mathrm{SA}}$, ${\mathrm{RA}}_{E\mathrm{tot}}^{\mathrm{SA}}$	5.2856 + 1.395 ${\mathrm{RA}}_{LogP}^{\mathrm{SA}}$ − 0.430 ${\mathrm{RA}}_{E\mathrm{tot}}^{\mathrm{SA}}$	${\mathrm{ARA}}_{LogP,E\mathrm{tot}}^{\mathrm{SA}}$	0.991

${\mathrm{RA}}_{\chi }^{\mathrm{SA}}$, ${\mathrm{RA}}_{\eta }^{\mathrm{SA}}$, ${\mathrm{RA}}_{POL}^{\mathrm{SA}}$	5.2856 + 0.852 ${\mathrm{RA}}_{\chi }^{\mathrm{SA}}$ + 0.176 ${\mathrm{RA}}_{\eta }^{\mathrm{SA}}$ − 0.030 ${\mathrm{RA}}_{POL}^{\mathrm{SA}}$	${\mathrm{ARA}}_{\chi ,\eta ,POL}^{\mathrm{SA}}$	0.999
${\mathrm{RA}}_{\chi }^{\mathrm{SA}}$, ${\mathrm{RA}}_{\eta }^{\mathrm{SA}}$, ${\mathrm{RA}}_{LogP}^{\mathrm{SA}}$	5.2856 + 0.884 ${\mathrm{RA}}_{\chi }^{\mathrm{SA}}$ + 0.123 ${\mathrm{RA}}_{\eta }^{\mathrm{SA}}$ − 0.007 ${\mathrm{RA}}_{LogP}^{\mathrm{SA}}$	${\mathrm{ARA}}_{\chi ,\eta ,LogP}^{\mathrm{SA}}$	0.999
${\mathrm{RA}}_{\chi }^{\mathrm{SA}}$, ${\mathrm{RA}}_{\eta }^{\mathrm{SA}}$, ${\mathrm{RA}}_{E\mathrm{tot}}^{\mathrm{SA}}$	5.2856 + 0.892 ${\mathrm{RA}}_{\chi }^{\mathrm{SA}}$ + 0.104 ${\mathrm{RA}}_{\eta }^{\mathrm{SA}}$ + 0.004 ${\mathrm{RA}}_{E\mathrm{tot}}^{\mathrm{SA}}$	${\mathrm{ARA}}_{\chi ,\eta ,E\mathrm{tot}}^{\mathrm{SA}}$	0.999
${\mathrm{RA}}_{\chi }^{\mathrm{SA}}$, ${\mathrm{RA}}_{POL}^{\mathrm{SA}}$, ${\mathrm{RA}}_{LogP}^{\mathrm{SA}}$	5.2856 + 0.941 ${\mathrm{RA}}_{\chi }^{\mathrm{SA}}$ − 0.045 ${\mathrm{RA}}_{POL}^{\mathrm{SA}}$ + 0.102 ${\mathrm{RA}}_{LogP}^{\mathrm{SA}}$	${\mathrm{ARA}}_{\chi ,POL,LogP}^{\mathrm{SA}}$	0.999
${\mathrm{RA}}_{\chi }^{\mathrm{SA}}$, ${\mathrm{RA}}_{POL}^{\mathrm{SA}}$, ${\mathrm{RA}}_{E\mathrm{tot}}^{\mathrm{SA}}$	5.2856 + 1.005 ${\mathrm{RA}}_{\chi }^{\mathrm{SA}}$ − 0.081 ${\mathrm{RA}}_{POL}^{\mathrm{SA}}$ + 0.080 ${\mathrm{RA}}_{E\mathrm{tot}}^{\mathrm{SA}}$	${\mathrm{ARA}}_{\chi ,POL,E\mathrm{tot}}^{\mathrm{SA}}$	0.999
${\mathrm{RA}}_{\chi }^{\mathrm{SA}}$, ${\mathrm{RA}}_{LogP}^{\mathrm{SA}}$, ${\mathrm{RA}}_{E\mathrm{tot}}^{\mathrm{SA}}$	5.2856 + 0.983 ${\mathrm{RA}}_{\chi }^{\mathrm{SA}}$ − 0.003 ${\mathrm{RA}}_{LogP}^{\mathrm{SA}}$ + 0.023 ${\mathrm{RA}}_{E\mathrm{tot}}^{\mathrm{SA}}$	${\mathrm{ARA}}_{\chi ,LogP,E\mathrm{tot}}^{\mathrm{SA}}$	0.999
${\mathrm{RA}}_{\eta }^{\mathrm{SA}}$, ${\mathrm{RA}}_{POL}^{\mathrm{SA}}$, ${\mathrm{RA}}_{LogP}^{\mathrm{SA}}$	5.2856 + 0.951 ${\mathrm{RA}}_{\eta }^{\mathrm{SA}}$ − 0.418 ${\mathrm{RA}}_{POL}^{\mathrm{SA}}$ + 0.450 ${\mathrm{RA}}_{LogP}^{\mathrm{SA}}$	${\mathrm{ARA}}_{\eta ,POL,LogP}^{\mathrm{SA}}$	0.997
${\mathrm{RA}}_{\eta }^{\mathrm{SA}}$, ${\mathrm{RA}}_{POL}^{\mathrm{SA}}$, ${\mathrm{RA}}_{E\mathrm{tot}}^{\mathrm{SA}}$	5.2856 + 1.212 ${\mathrm{RA}}_{\eta }^{\mathrm{SA}}$ − 0.290 ${\mathrm{RA}}_{POL}^{\mathrm{SA}}$ + 0.067 ${\mathrm{RA}}_{E\mathrm{tot}}^{\mathrm{SA}}$	${\mathrm{ARA}}_{\eta ,POL,E\mathrm{tot}}^{\mathrm{SA}}$	0.997
${\mathrm{RA}}_{\eta }^{\mathrm{SA}}$, ${\mathrm{RA}}_{LogP}^{\mathrm{SA}}$, ${\mathrm{RA}}_{E\mathrm{tot}}^{\mathrm{SA}}$	5.2856 + 0.955 ${\mathrm{RA}}_{\eta }^{\mathrm{SA}}$ + 0.213 ${\mathrm{RA}}_{LogP}^{\mathrm{SA}}$ − 0.185 ${\mathrm{RA}}_{E\mathrm{tot}}^{\mathrm{SA}}$	${\mathrm{ARA}}_{\eta ,LogP,E\mathrm{tot}}^{\mathrm{SA}}$	0.997
${\mathrm{RA}}_{POL}^{\mathrm{SA}}$, ${\mathrm{RA}}_{LogP}^{\mathrm{SA}}$, ${\mathrm{RA}}_{E\mathrm{tot}}^{\mathrm{SA}}$	5.2856 − 0.423 ${\mathrm{RA}}_{POL}^{\mathrm{SA}}$ + 1.617 ${\mathrm{RA}}_{LogP}^{\mathrm{SA}}$ − 0.227 ${\mathrm{RA}}_{E\mathrm{tot}}^{\mathrm{SA}}$	${\mathrm{ARA}}_{POL,LogP,E\mathrm{tot}}^{\mathrm{SA}}$	0.991
${\mathrm{RA}}_{\chi }^{\mathrm{SA}}$, ${\mathrm{RA}}_{\eta }^{\mathrm{SA}}$, ${\mathrm{RA}}_{POL}^{\mathrm{SA}}$, ${\mathrm{RA}}_{LogP}^{\mathrm{SA}}$	5.2856 + 0.815 ${\mathrm{RA}}_{\chi }^{\mathrm{SA}}$ + 0.170 ${\mathrm{RA}}_{\eta }^{\mathrm{SA}}$ − 0.082 ${\mathrm{RA}}_{POL}^{\mathrm{SA}}$ + 0.093 ${\mathrm{RA}}_{LogP}^{\mathrm{SA}}$	${\mathrm{ARA}}_{\chi ,\eta ,POL,LogP}^{\mathrm{SA}}$	0.999
${\mathrm{RA}}_{\chi }^{\mathrm{SA}}$, ${\mathrm{RA}}_{POL}^{\mathrm{SA}}$, ${\mathrm{RA}}_{LogP}^{\mathrm{SA}}$, ${\mathrm{RA}}_{E\mathrm{tot}}^{\mathrm{SA}}$	5.2856 + 0.966 ${\mathrm{RA}}_{\chi }^{\mathrm{SA}}$ − 0.121 ${\mathrm{RA}}_{POL}^{\mathrm{SA}}$ + 0.083 ${\mathrm{RA}}_{LogP}^{\mathrm{SA}}$ + 0.074 ${\mathrm{RA}}_{E\mathrm{tot}}^{\mathrm{SA}}$	${\mathrm{ARA}}_{\chi ,POL,LogP,E\mathrm{tot}}^{\mathrm{SA}}$	0.999
${\mathrm{RA}}_{\eta }^{\mathrm{SA}}$, ${\mathrm{RA}}_{POL}^{\mathrm{SA}}$, ${\mathrm{RA}}_{LogP}^{\mathrm{SA}}$, ${\mathrm{RA}}_{E\mathrm{tot}}^{\mathrm{SA}}$	5.2856 + 0.966 ${\mathrm{RA}}_{\eta }^{\mathrm{SA}}$ − 0.461 ${\mathrm{RA}}_{POL}^{\mathrm{SA}}$ + 0.422 ${\mathrm{RA}}_{LogP}^{\mathrm{SA}}$ + 0.038 ${\mathrm{RA}}_{E\mathrm{tot}}^{\mathrm{SA}}$	${\mathrm{ARA}}_{\eta ,POL,LogP,E\mathrm{tot}}^{\mathrm{SA}}$	0.999
${\mathrm{RA}}_{\chi }^{\mathrm{SA}}$, ${\mathrm{RA}}_{\eta }^{\mathrm{SA}}$, ${\mathrm{RA}}_{LogP}^{\mathrm{SA}}$, ${\mathrm{RA}}_{E\mathrm{tot}}^{\mathrm{SA}}$	5.2856 + 0.902 ${\mathrm{RA}}_{\chi }^{\mathrm{SA}}$ + 0.117 ${\mathrm{RA}}_{\eta }^{\mathrm{SA}}$ − 0.034 ${\mathrm{RA}}_{LogP}^{\mathrm{SA}}$ + 0.016 ${\mathrm{RA}}_{E\mathrm{tot}}^{\mathrm{SA}}$	${\mathrm{ARA}}_{\chi ,\eta ,LogP,E\mathrm{tot}}^{\mathrm{SA}}$	0.999
${\mathrm{RA}}_{\chi }^{\mathrm{SA}}$, ${\mathrm{RA}}_{\eta }^{\mathrm{SA}}$, ${\mathrm{RA}}_{POL}^{\mathrm{SA}}$, ${\mathrm{RA}}_{E\mathrm{tot}}^{\mathrm{SA}}$	5.2856 + 0.858 ${\mathrm{RA}}_{\chi }^{\mathrm{SA}}$ + 0.193 ${\mathrm{RA}}_{\eta }^{\mathrm{SA}}$ − 0.138 ${\mathrm{RA}}_{POL}^{\mathrm{SA}}$ + 0.088 ${\mathrm{RA}}_{E\mathrm{tot}}^{\mathrm{SA}}$	${\mathrm{ARA}}_{\chi ,\eta ,POL,E\mathrm{tot}}^{\mathrm{SA}}$	0.999
${\mathrm{RA}}_{\chi }^{\mathrm{SA}}$, ${\mathrm{RA}}_{\eta }^{\mathrm{SA}}$, ${\mathrm{RA}}_{POL}^{\mathrm{SA}}$, ${\mathrm{RA}}_{LogP}^{\mathrm{SA}}$, ${\mathrm{RA}}_{E\mathrm{tot}}^{\mathrm{SA}}$	5.2856 + 0.830 ${\mathrm{RA}}_{\chi }^{\mathrm{SA}}$ + 0.188 ${\mathrm{RA}}_{\eta }^{\mathrm{SA}}$ − 0.171 ${\mathrm{RA}}_{POL}^{\mathrm{SA}}$ + 0.070 ${\mathrm{RA}}_{LogP}^{\mathrm{SA}}$ + 0.083 ${\mathrm{RA}}_{E\mathrm{tot}}^{\mathrm{SA}}$	${\mathrm{ARA}}_{\chi ,\eta ,POL,LogP,E\mathrm{tot}}^{\mathrm{SA}}$	0.999

${\mathrm{RA}}_{\left(\chi ,\eta \right)}^{\mathrm{SA}}$	5.2856 + ${\mathrm{RA}}_{\left(\chi ,\eta \right)}^{\mathrm{SA}}$	${\mathrm{ARA}}_{\left(\chi ,\eta \right)}^{\mathrm{SA}}$	0.99
${\mathrm{RA}}_{\left(\chi ,POL\right)}^{\mathrm{SA}}$	5.2856 + ${\mathrm{RA}}_{\left(\chi ,POL\right)}^{\mathrm{SA}}$	${\mathrm{ARA}}_{\left(\chi ,POL\right)}^{\mathrm{SA}}$	0.96
${\mathrm{RA}}_{\left(\chi ,LogP\right)}^{\mathrm{SA}}$	5.2856 + ${\mathrm{RA}}_{\left(\chi ,LogP\right)}^{\mathrm{SA}}$	${\mathrm{ARA}}_{\left(\chi ,LogP\right)}^{\mathrm{SA}}$	0.985
${\mathrm{RA}}_{\left(\chi ,E\mathrm{tot}\right)}^{\mathrm{SA}}$	5.2856 + ${\mathrm{RA}}_{\left(\chi ,E\mathrm{tot}\right)}^{\mathrm{SA}}$	${\mathrm{ARA}}_{\left(\chi ,E\mathrm{tot}\right)}^{\mathrm{SA}}$	0.928
${\mathrm{RA}}_{\left(\eta ,POL\right)}^{\mathrm{SA}}$	5.2856 + ${\mathrm{RA}}_{\left(\eta ,POL\right)}^{\mathrm{SA}}$	${\mathrm{ARA}}_{\left(\eta ,POL\right)}^{\mathrm{SA}}$	0.985
${\mathrm{RA}}_{\left(\eta ,LogP\right)}^{\mathrm{SA}}$	5.2856 + ${\mathrm{RA}}_{\left(\eta ,LogP\right)}^{\mathrm{SA}}$	${\mathrm{ARA}}_{\left(\eta ,LogP\right)}^{\mathrm{SA}}$	0.985
${\mathrm{RA}}_{\left(\eta ,E\mathrm{tot}\right)}^{\mathrm{SA}}$	5.2856 + ${\mathrm{RA}}_{\left(\eta ,E\mathrm{tot}\right)}^{\mathrm{SA}}$	${\mathrm{ARA}}_{\left(\eta ,E\mathrm{tot}\right)}^{\mathrm{SA}}$	0.92
${\mathrm{RA}}_{\left(POL,LogP\right)}^{\mathrm{SA}}$	5.2856 + ${\mathrm{RA}}_{\left(POL,LogP\right)}^{\mathrm{SA}}$	${\mathrm{ARA}}_{\left(POL,LogP\right)}^{\mathrm{SA}}$	0.93
${\mathrm{RA}}_{\left(POL,E\mathrm{tot}\right)}^{\mathrm{SA}}$	5.2856 + ${\mathrm{RA}}_{\left(POL,E\mathrm{tot}\right)}^{\mathrm{SA}}$	${\mathrm{ARA}}_{\left(POL,E\mathrm{tot}\right)}^{\mathrm{SA}}$	0.923
${\mathrm{RA}}_{\left(E\mathrm{tot},LogP\right)}^{\mathrm{SA}}$	5.2856 + ${\mathrm{RA}}_{\left(E\mathrm{tot},LogP\right)}^{\mathrm{SA}}$	${\mathrm{ARA}}_{\left(E\mathrm{tot},LogP\right)}^{\mathrm{SA}}$	0.902

${\mathrm{RA}}_{\left(\chi ,\eta ),(POL,LogP\right)}^{\mathrm{SA}}$	5.2856 + 1.200 ${\mathrm{RA}}_{\left(\chi ,\eta \right)}^{\mathrm{SA}}$ − 0.212 ${\mathrm{RA}}_{\left(POL,LogP\right)}^{\mathrm{SA}}$	${\mathrm{ARA}}_{\left(\chi ,\eta ),(POL,LogP\right)}^{\mathrm{SA}}$	0.996
${\mathrm{RA}}_{\left(\chi ,\eta ),(POL,E\mathrm{tot}\right)}^{\mathrm{SA}}$	5.2856 + 1.0003 ${\mathrm{RA}}_{\left(\chi ,\eta \right)}^{\mathrm{SA}}$ − 0.0003 ${\mathrm{RA}}_{\left(POL,E\mathrm{tot}\right)}^{\mathrm{SA}}$	${\mathrm{ARA}}_{\left(\chi ,\eta )(POL,E\mathrm{tot}\right)}^{\mathrm{SA}}$	0.995
${\mathrm{RA}}_{\left(\chi ,\eta ),(E\mathrm{tot},LogP\right)}^{\mathrm{SA}}$	5.2856 + 0.9991 ${\mathrm{RA}}_{\chi ,\eta }^{\mathrm{SA}}$ + 0.0009 ${\mathrm{RA}}_{E\mathrm{tot},LogP}^{\mathrm{SA}}$	${\mathrm{ARA}}_{\left(\chi ,\eta ),(E\mathrm{tot},LogP\right)}^{\mathrm{SA}}$	0.995
${\mathrm{RA}}_{\left(\chi ,POL),(\eta ,LogP\right)}^{\mathrm{SA}}$	5.2856 − 0.490 ${\mathrm{RA}}_{\left(\chi ,POL\right)}^{\mathrm{SA}}$ + 1.471 ${\mathrm{RA}}_{\left(\eta ,LogP\right)}^{\mathrm{SA}}$	${\mathrm{ARA}}_{\left(\chi ,POL),(\eta ,LogP\right)}^{\mathrm{SA}}$	0.988
${\mathrm{RA}}_{\left(\chi ,POL),(\eta ,E\mathrm{tot}\right)}^{\mathrm{SA}}$	5.2856 + 1.033 ${\mathrm{RA}}_{\left(\chi ,POL\right)}^{\mathrm{SA}}$ − 0.036 ${\mathrm{RA}}_{\left(\eta ,E\mathrm{tot}\right)}^{\mathrm{SA}}$	${\mathrm{ARA}}_{\left(\chi ,POL),(\eta ,E\mathrm{tot}\right)}^{\mathrm{SA}}$	0.96
${\mathrm{RA}}_{\left(\chi ,POL),(E\mathrm{tot},LogP\right)}^{\mathrm{SA}}$	5.2856 + 0.945 ${\mathrm{RA}}_{\left(\chi ,POL\right)}^{\mathrm{SA}}$ + 0.062 ${\mathrm{RA}}_{(E\mathrm{tot},Log)P}^{\mathrm{SA}}$	${\mathrm{ARA}}_{\left(\chi ,POL),(E\mathrm{tot},LogP\right)}^{\mathrm{SA}}$	0.961
${\mathrm{RA}}_{\left(\chi ,LogP),(\eta ,POL\right)}^{\mathrm{SA}}$	5.2856 + 1.319 ${\mathrm{RA}}_{\left(\chi ,LogP\right)}^{\mathrm{SA}}$ − 0.339 ${\mathrm{RA}}_{\left(\eta ,POL\right)}^{\mathrm{SA}}$	${\mathrm{ARA}}_{\left(\chi ,LogP),(\eta ,POL\right)}^{\mathrm{SA}}$	0.987
${\mathrm{RA}}_{\left(\chi ,LogP),(\eta ,E\mathrm{tot}\right)}^{\mathrm{SA}}$	5.2856 + 1.148 ${\mathrm{RA}}_{\left(\chi ,LogP\right)}^{\mathrm{SA}}$ − 0.167 ${\mathrm{RA}}_{\left(\eta ,E\mathrm{tot}\right)}^{\mathrm{SA}}$	${\mathrm{ARA}}_{\left(\chi ,LogP),(\eta ,E\mathrm{tot}\right)}^{\mathrm{SA}}$	0.986
${\mathrm{RA}}_{\left(\chi ,LogP),(POL,E\mathrm{tot}\right)}^{\mathrm{SA}}$	5.2856 + 1.086 ${\mathrm{RA}}_{\left(\chi ,LogP\right)}^{\mathrm{SA}}$ − 0.097 ${\mathrm{RA}}_{\left(POL,E\mathrm{tot}\right)}^{\mathrm{SA}}$	${\mathrm{ARA}}_{\left(\chi ,LogP),(POL,Etot\right)}^{\mathrm{SA}}$	0.985
${\mathrm{RA}}_{\left(\chi ,E\mathrm{tot}),(\eta ,POL\right)}^{\mathrm{SA}}$	5.2856 − 0.084 ${\mathrm{RA}}_{\left(\chi ,E\mathrm{tot}\right)}^{\mathrm{SA}}$ + 1.081 ${\mathrm{RA}}_{\left(\eta ,POL\right)}^{\mathrm{SA}}$	${\mathrm{ARA}}_{\left(\chi ,E\mathrm{tot}),(\eta ,POL\right)}^{\mathrm{SA}}$	0.948
${\mathrm{RA}}_{\left(\chi ,E\mathrm{tot}),(\eta ,LogP\right)}^{\mathrm{SA}}$	5.2856 − 0.388 ${\mathrm{RA}}_{(\chi ,Eto)t}^{\mathrm{SA}}$ + 1.353 ${\mathrm{RA}}_{\left(\eta ,LogP\right)}^{\mathrm{SA}}$	${\mathrm{ARA}}_{\left(\chi ,E\mathrm{tot}),(\eta ,LogP\right)}^{\mathrm{SA}}$	0.99
${\mathrm{RA}}_{\left(\chi ,E\mathrm{tot}),(\eta ,LogP\right)}^{\mathrm{SA}}$	5.2856 + 0.308 ${\mathrm{RA}}_{\left(\chi ,E\mathrm{tot}\right)}^{\mathrm{SA}}$ + 0.706 ${\mathrm{RA}}_{\left(POL,LogP\right)}^{\mathrm{SA}}$	${\mathrm{ARA}}_{\left(\chi ,E\mathrm{tot}),(\eta ,LogP\right)}^{\mathrm{SA}}$	0.944
${\mathrm{RA}}_{\left(\eta ,POL),(E\mathrm{tot},LogP\right)}^{\mathrm{SA}}$	5.2856 + 0.870 ${\mathrm{RA}}_{\left(\eta ,POL\right)}^{\mathrm{SA}}$ + 0.145 ${\mathrm{RA}}_{\left(E\mathrm{tot},LogP\right)}^{\mathrm{SA}}$	${\mathrm{ARA}}_{\left(\eta ,POL),(E\mathrm{tot},LogP\right)}^{\mathrm{SA}}$	0.949
${\mathrm{RA}}_{\left(\eta ,LogP),(POL,E\mathrm{tot}\right)}^{\mathrm{SA}}$	5.2856 + 1.155 ${\mathrm{RA}}_{\left(\eta ,LogP\right)}^{\mathrm{SA}}$ − 0.173 ${\mathrm{RA}}_{\left(POL,E\mathrm{tot}\right)}^{\mathrm{SA}}$	${\mathrm{ARA}}_{\left(\eta ,LogP),(POL,Etot\right)}^{\mathrm{SA}}$	0.987
${\mathrm{RA}}_{\left(\eta ,E\mathrm{tot}),(POL,LogP\right)}^{\mathrm{SA}}$	5.2856 + 0.342 ${\mathrm{RA}}_{\left(\eta ,E\mathrm{tot}\right)}^{\mathrm{SA}}$ + 0.685 ${\mathrm{RA}}_{\left(POL,LogP\right)}^{\mathrm{SA}}$	${\mathrm{ARA}}_{\left(\eta ,E\mathrm{tot}),(POL,LogP\right)}^{\mathrm{SA}}$	0.947

${\mathrm{RA}}_{\left(\chi ,\eta ,POL\right)}^{\mathrm{SA}}$	5.2856 + ${\mathrm{RA}}_{\left(\chi ,\eta ,POL\right)}^{\mathrm{SA}}$	${\mathrm{ARA}}_{\left(\chi ,\eta ,POL\right)}^{\mathrm{SA}}$	0.948
${\mathrm{RA}}_{\left(\chi ,\eta ,LogP\right)}^{\mathrm{SA}}$	5.2856 + ${\mathrm{RA}}_{\left(\chi ,\eta ,LogP\right)}^{\mathrm{SA}}$	${\mathrm{ARA}}_{\left(\chi ,\eta ,LogP\right)}^{\mathrm{SA}}$	0.985
${\mathrm{RA}}_{\left(\chi ,\eta ,E\mathrm{tot}\right)}^{\mathrm{SA}}$	5.2856 + ${\mathrm{RA}}_{\left(\chi ,\eta ,E\mathrm{tot}\right)}^{\mathrm{SA}}$	${\mathrm{ARA}}_{\left(\chi ,\eta ,E\mathrm{tot}\right)}^{\mathrm{SA}}$	0.916
${\mathrm{RA}}_{\left(\chi ,POL,LogP\right)}^{\mathrm{SA}}$	5.2856 + ${\mathrm{RA}}_{\left(\chi ,POL,LogP\right)}^{\mathrm{SA}}$	${\mathrm{ARA}}_{\left(\chi ,POL,LogP\right)}^{\mathrm{SA}}$	0.941
${\mathrm{RA}}_{\left(\chi ,POL,E\mathrm{tot}\right)}^{\mathrm{SA}}$	5.2856 + ${\mathrm{RA}}_{\left(\chi ,POL,E\mathrm{tot}\right)}^{\mathrm{SA}}$	${\mathrm{ARA}}_{\left(\chi ,POL,E\mathrm{tot}\right)}^{\mathrm{SA}}$	0.91
${\mathrm{RA}}_{\left(\chi ,LogP,E\mathrm{tot}\right)}^{\mathrm{SA}}$	5.2856 + ${\mathrm{RA}}_{\left(\chi ,LogP,E\mathrm{tot}\right)}^{\mathrm{SA}}$	${\mathrm{ARA}}_{\left(\chi ,LogP,E\mathrm{tot}\right)}^{\mathrm{SA}}$	0.89
${\mathrm{RA}}_{\left(\eta ,POL,LogP\right)}^{\mathrm{SA}}$	5.2856 + ${\mathrm{RA}}_{\left(\eta ,POL,LogP\right)}^{\mathrm{SA}}$	${\mathrm{ARA}}_{\left(\eta ,POL,LogP\right)}^{\mathrm{SA}}$	0.927
${\mathrm{RA}}_{\left(\eta ,POL,E\mathrm{tot}\right)}^{\mathrm{SA}}$	5.2856 + ${\mathrm{RA}}_{\left(\eta ,POL,E\mathrm{tot}\right)}^{\mathrm{SA}}$	${\mathrm{ARA}}_{\left(\eta ,POL,E\mathrm{tot}\right)}^{\mathrm{SA}}$	0.919
${\mathrm{RA}}_{\left(\eta ,LogP,E\mathrm{tot}\right)}^{\mathrm{SA}}$	5.2856 + ${\mathrm{RA}}_{\left(\eta ,LogP,E\mathrm{tot}\right)}^{\mathrm{SA}}$	${\mathrm{ARA}}_{\left(\eta ,LogP,E\mathrm{tot}\right)}^{\mathrm{SA}}$	0.899
${\mathrm{RA}}_{\left(POL,LogP,E\mathrm{tot}\right)}^{\mathrm{SA}}$	5.2856 + ${\mathrm{RA}}_{\left(POL,LogP,E\mathrm{tot}\right)}^{\mathrm{SA}}$	${\mathrm{ARA}}_{\left(POL,LogP,E\mathrm{tot}\right)}^{\mathrm{SA}}$	0.902

**Table 6.** Residual-alert QSARs for the models of Table 5 that fulfill Equation (10) with highest trial correlation factors. These are compared with the respective direct structural alert models of Table 3 using their correlation performances for the trial and test molecules in Tables 1 and 2, respectively.

**No. Crt.**	**Variabile**	**QSAR Model**	**R_trial_**	**R_test_**
*I_a_*	*χ^SA^*, $E_{\mathrm{tot}}^{\mathrm{SA}}$	*ARA^SA^* = 158.7 − 34*χ^SA^* − 0.003 $E_{\mathrm{tot}}^{\mathrm{SA}}$	0.368	0.168
		*A^SA^* = 5.58 − 0.255*χ^SA^* − 0.000032 $E_{\mathrm{tot}}^{\mathrm{SA}}$	0.371	0.127

*I_b_*	*η^SA^*, *POL^SA^*	*ARA^SA^* = −77.866 + 14.75*η^SA^* + 0.833*POL^SA^*	0.078	0.505
		*A^SA^* = 2.43 + 0.38*η^SA^* + 0.08*POL^SA^*	0.316	0.043

*I_c_*	*η^SA^*, *LogP^SA^*	*ARA^SA^* = −408.2 + 82*η^SA^* + 8*LogP^SA^*	0.063	0.725
		*A^SA^* = 4.75 + 0.05*η^SA^* + 0.179*LogP^SA^*	0.167	0.052

*I_d_*	*η^SA^*, $E_{\mathrm{tot}}^{\mathrm{SA}}$	*ARA^SA^* = −64.769 + 12.615*η^SA^* − 0.0023 $E_{\mathrm{tot}}^{\mathrm{SA}}$	0.384	0.087
		*A^SA^* = 2.6 + 0.314*η^SA^* − 0.00004 $E_{\mathrm{tot}}^{\mathrm{SA}}$	0.38	0.131

*I_e_*	*POL^SA^*, *LogP^SA^*	*ARA^SA^* = 2.76 + 1.4*POL^SA^* − 10.68*LogP^SA^*	0.040	0.222
		*A^SA^* = 4.36 + 0.12*POL^SA^*−0.44*LogP^SA^*	0.337	0.357

*I_f_*	*POL^SA^*, $E_{\mathrm{tot}}^{\mathrm{SA}}$	*ARA^SA^* = 19.821 − 2.928*POL^SA^* − 0.0071 $E_{\mathrm{tot}}^{\mathrm{SA}}$	0.369	0.015
		*A^SA^* = 4.38 − 0.0625*POL^SA^* − 0.00005 $E_{\mathrm{tot}}^{\mathrm{SA}}$	0.382	0.132

*I_g_*	*LogP^SA^*, $E_{\mathrm{tot}}^{\mathrm{SA}}$	*ARA^SA^* = 3.142 − 6.314*LogP^SA^* − 0.0028 $E_{\mathrm{tot}}^{\mathrm{SA}}$	0.386	0.016
		*A^SA^* = 4.09 − 0.412*LogP^SA^* − 0.00006 $E_{\mathrm{tot}}^{\mathrm{SA}}$	0.430	0.007

${\mathrm{II}}_{a}^{1}={\mathrm{II}}_{f}^{1}$	*χ^SA^*, *POL^SA^*, $E_{\mathrm{tot}}^{\mathrm{SA}}$	*ARA^SA^* = 84 − 17.5*χ^SA^* − *POL^SA^* − 0.005 $E_{\mathrm{tot}}^{\mathrm{SA}}$	0.373	0.056
		*A^SA^* = 6.46 − 0.45*χ^SA^* − 0.092*POL^SA^* − 0.0007 $E_{\mathrm{tot}}^{\mathrm{SA}}$	0.414	0.018

${\mathrm{II}}_{a}^{2}$	*χ^SA^*, *LogP^SA^*, $E_{\mathrm{tot}}^{\mathrm{SA}}$	*ARA^SA^* = 108 − 22.66*χ^SA^* − 0.133*LogP^SA^* − 0.0023 $E_{\mathrm{tot}}^{\mathrm{SA}}$	0.371	0.149
		*A^SA^* = 5.75 − 0.36*χ^SA^* − 0.45*LogP^SA^* − 0.00006 $E_{\mathrm{tot}}^{\mathrm{SA}}$	0.451	0.136

${\mathrm{II}}_{b}^{1}$	*χ^SA^*, *η^SA^*, *POL^SA^*	*ARA^SA^* = −210 + 29.5*χ^SA^* + 13*η^SA^* + 0.5*POL^SA^*	0.018	0.592
		*A^SA^* = 2.62 − 0.035*χ^SA^* + 0.382*η^SA^* + 0.08*POL^SA^*	0.31	0.027

${\mathrm{II}}_{b}^{2}={\mathrm{II}}_{e}^{2}$	*η^SA^*, *POL^SA^*, *LogP^SA^*	*ARA^SA^* = −44.705 + 8.294*η^SA^* + 1.176*POL^SA^* − 4.467*LogP^SA^*	0.122	0.286
		*A^SA^* = 2.05 + 0.39*η^SA^* + 0.16*POL^SA^* − 0.449*LogP^SA^*	0.373	0.112

${\mathrm{II}}_{b}^{3}={\mathrm{II}}_{d}^{1}={\mathrm{II}}_{f}^{2}$	*η^SA^*, *POL^SA^*, $E_{\mathrm{tot}}^{\mathrm{SA}}$	*ARA^SA^* = −85.72 + 16.363*η^SA^* + 1.272*POL^SA^* + 0.0018 $E_{\mathrm{tot}}^{\mathrm{SA}}$	0.304	0.178
		*A^SA^* = 3.09 + 0.22*η^SA^* − 0.034*POL^SA^* − 0.00005 $E_{\mathrm{tot}}^{\mathrm{SA}}$	0.392	0.152

${\mathrm{II}}_{c}={\mathrm{II}}_{d}^{2}$	*η^SA^*, *LogP^SA^*, $E_{\mathrm{tot}}^{\mathrm{SA}}$	*ARA^SA^* = −42.58 + 8.352*η^SA^* − 1.941*LogP^SA^* − 0.0029 $E_{\mathrm{tot}}^{\mathrm{SA}}$	0.382	0.039
		*A^SA^* = 3.23 + 0.156*η^SA^* − 0.368*LogP^SA^* − 0.0006 $E_{\mathrm{tot}}^{\mathrm{SA}}$	0.436	0.012

${\mathrm{II}}_{e}^{1}$	*χ^SA^*, *POL^SA^*, *LogP^SA^*	*ARA^SA^* = −154 + 32.5*χ^SA^* + *POL^SA^* − 8*LogP^SA^*	0.019	0.399
		*A^SA^* = 4.52 − 0.0325*χ^SA^* + 0.12*POL^SA^* − 0.439*LogP^SA^*	0.337	0.370

${\mathrm{II}}_{e}^{3}={\mathrm{II}}_{f}^{3}$	*POL^SA^*, *LogP^SA^*, $E_{\mathrm{tot}}^{\mathrm{SA}}$	*ARA^SA^* = 2.151 + 0.636*POL^SA^* − 7.787*LogP* − 0.0021 $E_{\mathrm{tot}}^{\mathrm{SA}}$	0.394	0.038
		*A^SA^* = 4.07 + 0.015*POL^SA^* − 0.45*LogP^SA^* − 0.000057 $E_{\mathrm{tot}}^{\mathrm{SA}}$	0.431	0.034

*III*_1_	*χ^SA^*, *η^SA^*, *POL^SA^*, *LogP^SA^*	*ARA^SA^* = 42 − 2.052*χ^SA^* − 3.842*η^SA^* + 12.894*POL^SA^* − 13.842*LogP^SA^*	0.289	0.277
		*A^SA^* = 1.921 + 0.24*χ^SA^* + 0.4*η^SA^* + 0.16*POL^SA^* − 0.45*LogP^SA^*	0.373	0.007

*III*_2_	*χ^SA^*, *POL^SA^*, *LogP^SA^*, $E_{\mathrm{tot}}^{\mathrm{SA}}$	*ARA^SA^* = −34.928 − 5.571*χ^SA^* + 6*POL^SA^* − 22.142*LogP^SA^* − 0.0006 $E_{\mathrm{tot}}^{\mathrm{SA}}$	0.372	0.296
		*A^SA^* = 5.894 − 0.39*χ^SA^* − 0.016*POL^SA^* − 0.41*LogP^SA^* − 0.00007 $E_{\mathrm{tot}}^{\mathrm{SA}}$	0.452	0.106

*III*_3_	*η^SA^*, *POL^SA^*, *LogP^SA^*, $E_{\mathrm{tot}}^{\mathrm{SA}}$	*ARA^SA^* = −19.228 + 4.085*η^SA^* + 0.657*POL^SA^* − 1.914*LogP^SA^* + 0.0002 $E_{\mathrm{tot}}^{\mathrm{SA}}$	0.21	0.184
		*A^SA^* = 2.72 + 0.23*η^SA^* + 0.04*POL^SA^* − 0.45*LogP^SA^* − 0.00005 $E_{\mathrm{tot}}^{\mathrm{SA}}$	0.441	0.033

*III*_4_	*χ^SA^*, *η^SA^*, *LogP^SA^*, $E_{\mathrm{tot}}^{\mathrm{SA}}$	*ARA^SA^* = 384 − 63*χ^SA^* − 17*η^SA^* − 5*LogP^SA^* − 0.004 $E_{\mathrm{tot}}^{\mathrm{SA}}$	0.348	0.300
		*A^SA^* = 4.94 − 0.35*χ^SA^* + 0.13*η^SA^* − 0.41*LogP^SA^*^−^ 0.00006 $E_{\mathrm{tot}}^{\mathrm{SA}}$	0.455	0.066

*III*_5_	*χ^SA^*, *η^SA^*, *POL^SA^*, $E_{\mathrm{tot}}^{\mathrm{SA}}$	*ARA^SA^* = 36.66 + 27.33*χ^SA^* + 3.666*η^SA^* − 172*POL^SA^* − 0.0003 $E_{\mathrm{tot}}^{\mathrm{SA}}$	0.274	0.225
		*A^SA^* = 5.587 − 0.41*χ^SA^* + 0.12*η^SA^* − 0.07*POL^SA^* − 0.00006 $E_{\mathrm{tot}}^{\mathrm{SA}}$	0.416	0.021

**Table 7.** Trial-test averages of the correlations’ connected paths between the endpoint models of Table 6, computed using the Euler Equation (11).

**Endpoint Paths**	Δ*R_ARA^SA^_*			Δ*R_A^SA^_*		

	**Trial**	**Test**	**Average**	**Trial**	**Test**	**Average**
$I_{a}\to {\mathrm{II}}_{a}^{1}={\mathrm{II}}_{f}^{1}\to {\mathrm{III}}_{2}$	0.005099	0.264847	0.134973	0.057384	0.140089	0.098736
$I_{a}\to {\mathrm{II}}_{a}^{2}\to {\mathrm{III}}_{2}$	0.003162	0.148222	**0.075692^α^**	0.080006	0.031320	**0.055663^β^**
$I_{a}\to {\mathrm{II}}_{a}^{2}\to {\mathrm{III}}_{4}$	0.023194	0.152190	0.087692	0.080099	0.070576	0.075337

$I_{b}\to {\mathrm{II}}_{b}^{2}={\mathrm{II}}_{e}^{2}\to {\mathrm{III}}_{3}$	0.098386	0.241588	0.169987	0.088729	0.104890	0.09681
$I_{b}\to {\mathrm{II}}_{b}^{3}={\mathrm{II}}_{d}^{1}={\mathrm{II}}_{f}^{2}\to {\mathrm{III}}_{3}$	0.244769	0.327055	0.285912	0.090426	0.161375	0.125901

$I_{c}\to {\mathrm{II}}_{b}^{2}={\mathrm{II}}_{e}^{2}\to {\mathrm{III}}_{3}$	0.105948	0.450693	0.278321	0.216933	0.099201	0.158067
$I_{c}\to {\mathrm{II}}_{b}^{2}={\mathrm{II}}_{e}^{2}\to {\mathrm{III}}_{1}$	0.177115	0.439092	0.308104	0.206000	0.120933	0.163467
$I_{c}\to {\mathrm{II}}_{c}={\mathrm{II}}_{d}^{2}\to {\mathrm{III}}_{3}$	0.362415	0.701156	0.531786	0.269046	0.045177	0.157112
$I_{c}\to {\mathrm{II}}_{c}={\mathrm{II}}_{d}^{2}\to {\mathrm{III}}_{4}$	0.320806	0.733973	0.52739	0.269670	0.067201	0.168436

$I_{d}\to {\mathrm{II}}_{b}^{3}={\mathrm{II}}_{d}^{1}\to {\mathrm{III}}_{3}$	0.123434	0.091197	0.107316	0.050447	0.120838	0.085643
$I_{d}\to {\mathrm{II}}_{b}^{3}={\mathrm{II}}_{d}^{1}\to {\mathrm{III}}_{5}$	0.085440	0.102420	**0.09393^β^**	0.026832	0.132672	**0.079752^γ^**
$I_{d}\to {\mathrm{II}}_{c}={\mathrm{II}}_{d}^{2}\to {\mathrm{III}}_{3}$	0.172011	0.152738	0.162375	0.056222	0.120838	0.08853
$I_{d}\to {\mathrm{II}}_{c}={\mathrm{II}}_{d}^{2}\to {\mathrm{III}}_{4}$	0.034058	0.265377	0.149718	0.059135	0.130678	0.094907

$I_{e}\to {\mathrm{II}}_{b}^{2}={\mathrm{II}}_{e}^{2}\to {\mathrm{III}}_{3}$	0.120282	0.120415	**0.120349^γ^**	0.077369	0.257421	0.167395
$I_{e}\to {\mathrm{II}}_{e}^{3}={\mathrm{II}}_{f}^{3}\to {\mathrm{III}}_{3}$	0.431226	0.187882	0.309554	0.094530	0.323154	0.208842

$I_{f}\to {\mathrm{II}}_{b}^{3}={\mathrm{II}}_{d}^{1}={\mathrm{II}}_{f}^{2}\to {\mathrm{III}}_{3}$	0.114284	0.163110	0.138697	0.050009	0.120668	0.085339
$I_{f}\to {\mathrm{II}}_{e}^{3}={\mathrm{II}}_{f}^{3}\to {\mathrm{III}}_{3}$	0.185690	0.147800	0.166745	0.050009	0.098005	0.074007

$I_{g}\to {\mathrm{II}}_{a}^{2}={\mathrm{II}}_{g}^{1}\to {\mathrm{III}}_{4}$	0.027459	0.201221	0.11434	0.021377	0.146768	0.084073
$I_{g}\to {\mathrm{II}}_{c}={\mathrm{II}}_{d}^{2}\to {\mathrm{III}}_{3}$	0.172046	0.146812	0.159429	0.007810	0.021587	**0.014699^α^**
$I_{g}\to {\mathrm{II}}_{c}={\mathrm{II}}_{d}^{2}\to {\mathrm{III}}_{4}$	0.034234	0.262011	0.148123	0.019924	0.054230	0.037077
$I_{g}\to {\mathrm{II}}_{e}^{3}={\mathrm{II}}_{f}^{3}\to {\mathrm{III}}_{3}$	0.184173	0.147648	0.165911	0.010049	0.027018	0.018534
